# The CD6/ALCAM pathway promotes lupus nephritis via T cell–mediated responses

**DOI:** 10.1172/JCI147334

**Published:** 2022-01-04

**Authors:** Samantha A. Chalmers, Rajalakshmy Ayilam Ramachandran, Sayra J. Garcia, Evan Der, Leal Herlitz, Jeanette Ampudia, Dalena Chu, Nicole Jordan, Ting Zhang, Ioannis Parodis, Iva Gunnarsson, Huihua Ding, Nan Shen, Michelle Petri, Chi Chiu Mok, Ramesh Saxena, Krishna R. Polu, Stephen Connelly, Cherie T. Ng, Chandra Mohan, Chaim Putterman

**Affiliations:** 1 Division of Rheumatology, Department of Microbiology and Immunology, Albert Einstein College of Medicine, Bronx, New York, USA.; 2 Department of Biomedical Engineering, University of Houston, Houston, Texas, USA.; 3 Department of Pathology, Cleveland Clinic, Cleveland, Ohio, USA.; 4 Equillium, La Jolla, California, USA.; 5 Division of Rheumatology, Department of Medicine Solna, Karolinska Institute and Department of Gastroenterology, Dermatology and Rheumatology, Karolinska University Hospital, Stockholm, Sweden.; 6 Shanghai Jiao Tong University School of Medicine, Shanghai, China.; 7 Division of Rheumatology, Johns Hopkins University School of Medicine, Baltimore, Maryland, USA.; 8 Tuen Men Hospital, Hong Kong, China.; 9 Division of Nephrology, University of Texas Southwestern Medical Center at Dallas, Dallas, Texas, USA.; 10 Azrieli Faculty of Medicine, Bar-Ilan University, Safed, Israel.; 11 Research Institute, Galilee Medical Center, Nahariya, Israel.

**Keywords:** Autoimmunity, Lupus

## Abstract

T cells are central to the pathogenesis of lupus nephritis (LN), a common complication of systemic lupus erythematosus (SLE). CD6 and its ligand, activated leukocyte cell adhesion molecule (ALCAM), are involved in T cell activation and trafficking. Previously, we showed that soluble ALCAM is increased in urine (uALCAM) of patients with LN, suggesting that this pathway contributes to disease. To investigate, uALCAM was examined in 1038 patients with SLE and LN from 5 ethnically diverse cohorts; CD6 and ALCAM expression was assessed in LN kidney cells; and disease contribution was tested via antibody blockade of CD6 in murine models of SLE and acute glomerulonephritis. Extended cohort analysis offered resounding validation of uALCAM as a biomarker that distinguishes active renal involvement in SLE, irrespective of ethnicity. ALCAM was expressed by renal structural cells whereas CD6 expression was exclusive to T cells, with elevated numbers of CD6^+^ and ALCAM^+^ cells in patients with LN. CD6 blockade in models of spontaneous lupus and immune-complex glomerulonephritis revealed significant decreases in immune cells, inflammatory markers, and disease measures. Our data demonstrate the contribution of the CD6/ALCAM pathway to LN and SLE, supporting its use as a disease biomarker and therapeutic target.

## Introduction

Systemic lupus erythematosus (SLE) is a chronic autoimmune inflammatory disease that predominantly affects women in the reproductive age range. Approximately 50% of patients with SLE experience lupus nephritis (LN), a serious complication of SLE, which is accompanied by significant morbidity and mortality. Despite the use of potent antiinflammatory and immunosuppressive treatments, progression of LN to chronic kidney disease is common, and 10% of patients with LN develop end-stage kidney disease, a condition associated with a very poor prognosis (1).

Though the precise etiology of SLE is not well understood, the disease is broadly characterized by immune dysregulation, including aberrant T and B cell activity (2). Recent evidence has demonstrated that T cells, both CD4 and CD8, play a central role in the pathogenesis of both SLE and LN by mediating tissue damage and enhancing the production of autoantibodies by promoting B cell differentiation, proliferation, and maturation (3). T cells represent the majority of renal-infiltrating immune cells (4). Multiple CD4^+^ T helper (Th) cell subsets, including Th1, Th2, and Th17 and their associated cytokines, as well as cytotoxic CD8^+^ T cells, have all been implicated in the immune pathogenesis of both SLE and LN, highlighting the complex nature of the disease. Recent research has focused on Th17 cells as emerging key targets. High levels of IL‑17 predict poor histopathological outcome after immunosuppressive therapy in patients with LN (3, 5, 6), while elevated levels of Th17 cells are accompanied by a decrease in Tregs, suggesting that loss of functional immune balance may be involved in the pathogenesis of renal injury in patients with SLE (6).

T cells express CD6, a costimulatory membrane glycoprotein that is involved in effector T cell (Teff) activity and trafficking. The receptor has been implicated in multiple autoimmune and inflammatory diseases, including psoriasis, multiple sclerosis, rheumatoid arthritis, graft versus host disease, Sjögren’s syndrome, and inflammatory bowel disease (7–10). CD6 expression is primarily limited to T cells, with the highest expression on CD4^+^ T cells and more moderate expression on CD8^+^ T cells (11). Conversely, Tregs, as defined by FOXP3^+^, demonstrate low surface expression of CD6 (12). CD6 has 2 ligands, CD166/activated leukocyte cell adhesion molecule (ALCAM) (13) and CD318, also known as CUB domain containing protein 1 (CDCP1) (14). Here, we focus on the relationship of CD6 to ALCAM, a member of the immunoglobulin superfamily that is widely expressed in various tissues, including endothelial and epithelial cells, and on antigen-presenting cells (APCs).

The CD6/ALCAM pathway has several roles in T cell activation and function. First, CD6 colocalizes with the TCR/CD3 complex and binds ALCAM expressed on APCs. This strengthens the immune synapse to enhance and lengthen the time of T cell/APC interaction, which is necessary for optimal activation (15–18). Second, stimulation of CD6 via binding of ALCAM results in the recruitment of signaling factors such as SLP-76 and GADs that enhance TCR signaling, and in the activation of several mitogen-activated protein kinase (MAPK) pathways related to T cell activation, proliferation, and differentiation (19–25). Third, CD6 contributes to the infiltration of Teffs into inflamed tissues (26, 27). ALCAM is expressed in a variety of tissues, including the kidney, blood brain barrier, skin, lung, and gut, and has been associated with inflammatory diseases (28–31). In the presence of the proinflammatory cytokines such as TNF-α and IFN-γ, tissue expression of ALCAM is upregulated, which correlates with increased infiltration of CD6^+^ Teffs (10).

Recently, based on a comprehensive screen of more than 1000 urine proteins, we identified soluble urinary ALCAM (uALCAM) as one of only a few molecules that were elevated in the urine of patients with SLE with active renal involvement compared with patients with quiescent or no prior nephritis (32–34). This observation spurred several questions: (a) relevance to a larger population spanning multiple ethnicities, (b) involvement of the CD6/ALCAM pathway in disease mechanisms and pathology, and (c) whether this pathway is an appropriate target for disease monitoring or therapy. Consequently, in this study, we investigated the association of the CD6/ALCAM pathway with human disease by expanding the SLE and LN patient data sets to include more than 1000 individuals from 4 ethnicities and identifying the specific cell types within LN kidneys that express CD6 and ALCAM. Then, to establish an essential role for CD6/ALCAM interactions in disease pathogenesis, we investigated the effect of CD6 blockade in the MRL/lpr model of spontaneous SLE and LN and the nephrotoxic serum nephritis model of acute glomerulonephritis.

## Results

### Soluble uALCAM correlates with disease severity in patients with LN.

**Figure 1:**
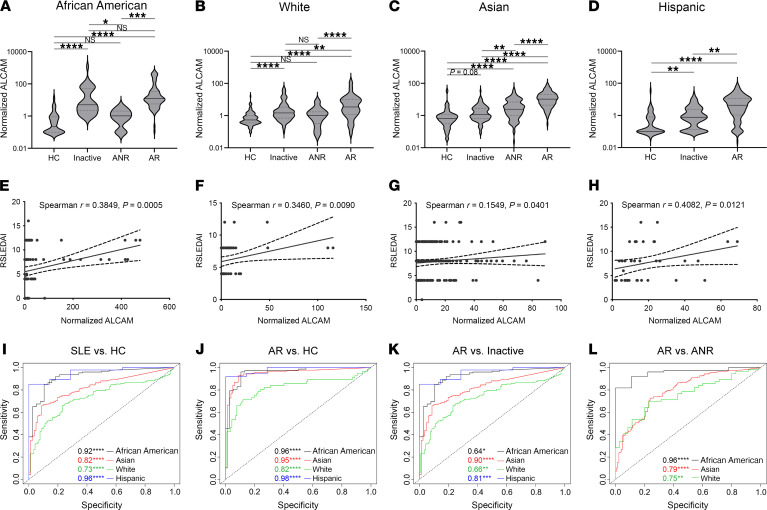
Soluble uALCAM is elevated in subjects with active renal disease and correlates with disease severity. uALCAM was assayed in 1038 individuals drawn from 4 ethnicities and 5 patient cohorts, as detailed in Supplemental Table 1, and normalized by urine creatinine levels. The demographics for the new patients included in this study are listed in Supplemental Table 2. The information pertaining to the other patients have already been published (31–33). (**A**–**D**) uALCAM levels differentiate disease states (healthy control [HC], inactive SLE [Inactive], active nonrenal SLE [ANR], active renal SLE [AR]) across multiple ethnicities: African American (**A**), White (**B**), Asian (**C**), and Hispanic (**D**). Data are presented as violin plots. (**E**–**G**) Correlation of uALCAM with sum of the renal scores of the rSLEDAI, a clinical measure of LN activity and damage, in African American (**E**), White (**F**), Asian (**G**), and Hispanic (**H**) patients. (**I**–**L**) ROC curves depicting the performance of uALCAM levels as a marker of disease state. The following comparisons were made: SLE vs. HC (**I**), AR vs. HC (**J**), AR vs. Inactive (**K**), AR vs. ANR (**L**). Comparisons between groups were performed using the Kruskal-Wallis test, while the correlation analysis was performed using Spearman correlation. ****P < 0.0001; ***P < 0.001; **P < 0.01; *P < 0.05.

To examine elevated uALCAM levels across an ethnically diverse set of patients, we collected data from 5 independent data sets of patients with SLE to form an extended cross-sectional cohort comprised of more than 1000 individuals (Supplemental Table 1; supplemental material available online with this article; https://doi.org/10.1172/JCI147334DS1). In all studies, uALCAM was normalized to urine creatinine levels, an analyte excreted at a relatively constant rate in urine among different individuals, to correct for differences due to dilution by urine volume. Patients with active LN exhibited significantly elevated levels of creatinine-normalized uALCAM, compared with patients with SLE with active nonrenal disease or inactive SLE, in all ethnic groups examined, as plotted for people who are African American, White, Asian, and Hispanic (Figure 1, A–D, respectively). Additionally, patients with active nonrenal SLE or inactive SLE exhibited intermediate levels of uALCAM, with these being higher than the levels observed in healthy controls. Further, uALCAM levels were significantly correlated with urine levels of TNF-α and IFN-γ (Supplemental Figure 1), inflammatory cytokines shown to increase cellular expression of ALCAM (27). Comparatively, assessment of the alternate CD6 ligand, CD318, showed urine levels that were below detection (data not shown) while serum levels did not distinguish between patients with active LN versus patients with active, nonrenal disease or inactive SLE (Supplemental Figure 2A).

Among the African American, White, and Hispanic patients with LN, uALCAM levels correlated significantly with the renal domain scores of the Systemic Lupus Erythematosus Disease Activity Index (rSLEDAI), a clinical measure of renal disease activity, with correlation coefficients ranging from 0.35 to 0.41 (Figure 1, E–H). uALCAM levels were able to distinguish active LN from active nonrenal lupus in all ethnic groups with ROC AUC values ranging from 0.75 in White patients to 0.96 in African American patients (Figure 1, I–L). African American patients exhibited increased absolute levels of creatinine-normalized uALCAM levels compared with patients of other ethnicities (Figure 1, A–D), as we previously reported (32). Thus, these extended studies offer resounding validation of uALCAM as a biomarker that distinguishes active renal involvement in SLE, irrespective of the patient’s ethnicity.

### Renal expression of CD6 and ALCAM is elevated in patients with LN.

**Figure 2:**
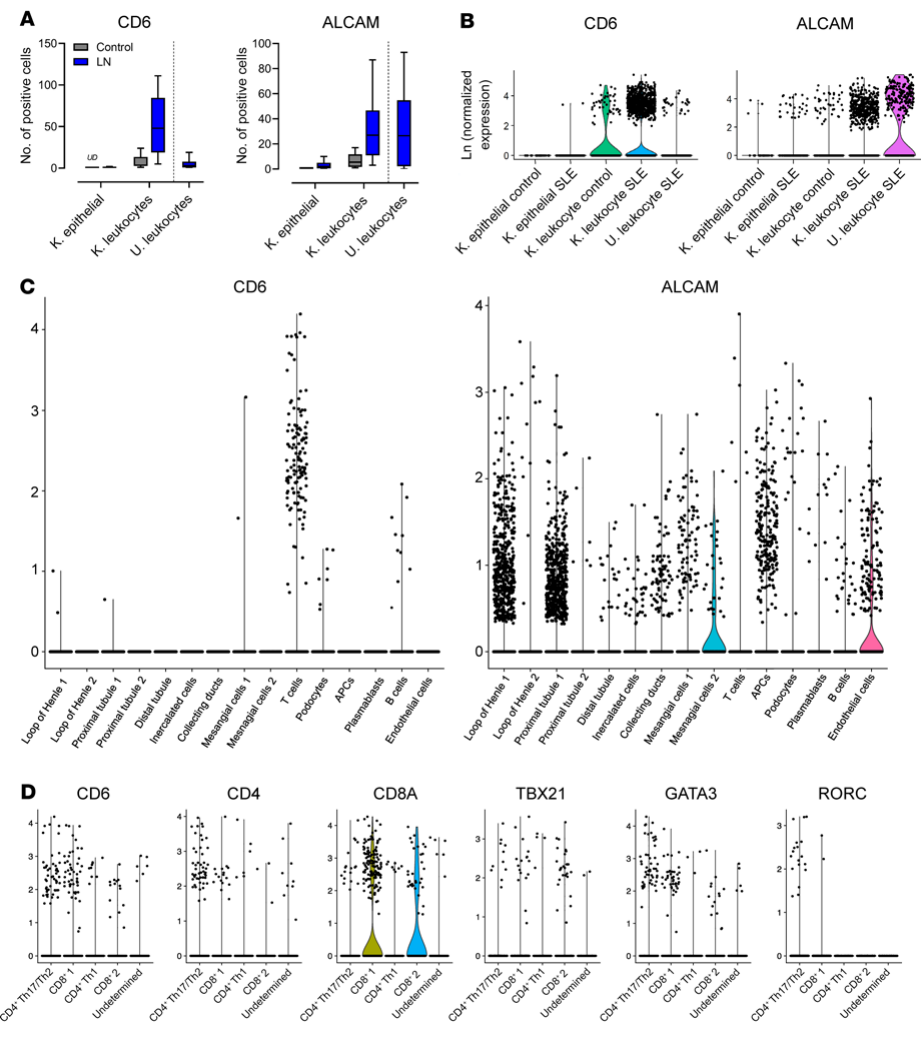
CD6 and ALCAM are overexpressed in renal tissue of patients with LN. A single-cell RNA-Seq data set derived from epithelial cells and leukocytes isolated from the kidney biopsies of 24 patients with LN and 9 control subjects and urine cells collected from patients with LN only was analyzed for CD6 and ALCAM expression. Cells from the patients of each respective disease state were combined for analysis. (**A**) Box and whisker plots representing 25th, 50th, and 75th percentiles ± min/max of the number of cells with detectable CD6 expression (left panel) and ALCAM expression (right panel) in LN and control. *UD* = undetected. Comparisons between groups performed by Mann-Whitney *U* test. **P < 0.01 for CD6 expression on K. leukocytes. (**B**) Single-cell expression levels of CD6 and ALCAM. (**C**) Violin plots depicting expression of CD6 (left panel) and ALCAM (right panel) within specific renal cell populations of patients with LN. Expression of CD6 is primarily elevated in T cells and expression of ALCAM is elevated in epithelial cells isolated from renal tissue of LN subjects. (**D**) Violin plots of coexpression of CD6 with markers of CD4, CD8, and T helper subsets, Th1 (TBX21), Th2 (GATA3), and Th17 (RORC) subsets.

The correlation between uALCAM levels and disease status suggests that the CD6/ALCAM pathway is a key driver of disease. To investigate the presence of CD6 and ALCAM on renal cell populations in LN, we utilized single-cell RNA-Seq data sets derived from the renal tissue of patients with LN as part of the Accelerated Medicines Partnership (35). Data from renal biopsies of 24 patients with LN and 9 healthy controls (Supplemental Table 3) were analyzed specifically for expression of *CD6* and *ALCAM* on renal leukocyte and structural cell populations (Figure 2). When compared with non-SLE control subjects, patients with LN had increased numbers of *CD6*-expressing leukocytes and *ALCAM*-expressing leukocytes and epithelial cells in the kidney (Figure 2A). Mean expression level in positive cells did not appear to differ between patients with LN and control subjects for both *CD6* and *ALCAM* (Figure 2B), highlighting that increased *CD6* and *ALCAM* in tissue is primarily due to increased numbers of expressing cells. We did additionally note that *ALCAM* expression levels appeared to be slightly higher in urine leukocytes compared with renal tissue leukocytes in patients with LN (Figure 2B, right panel). Expression in urine cells could not be compared with control subjects as leukocytes are not normally present in urine, but patients with LN had both *CD6*-expressing and *ALCAM*-expressing leukocytes in their urine (Figure 2, A and B).

To further understand CD6 and ALCAM expression patterns in patients with LN, we used cell-clustering analysis to define leukocyte cell types (T cells, B cells, APCs) and structural renal cell types (podocytes, endothelial cells, proximal tubule cells, loop of Henle cells, mesangial cells, etc.; Figure 2C). *CD6* was primarily expressed on T cells in a manner consistent with other reports of CD6 expression (Figure 2C and ref. 36). Patients with class III or IV LN trended toward having more CD6-expressing cells than controls. Within T cell populations, CD6 expression was associated with both CD4 and CD8, and with *TBX21* (Th1), *GATA3* (Th2), and *RORC* (Th17) expressing T cells (Figure 2D). *ALCAM* was strongly expressed on a number of resident and infiltrating renal cells including the Loop of Henle, proximal tubules, endothelial cells, and APCs (Figure 2C) and detected in more moderate numbers on mesangial cells, podocytes, and B cells. A summary of renal cell expression profiles for both *CD6* and *ALCAM* is presented in Table 1. There waslittle detection of *CDCP1* (CD318 gene) expression in renal leukocyte populations, and tubular, mesangial, and endothelial cells (Supplemental Figure 2B). Furthermore, CD3 stimulation of PBMCs paired with costimulation of ALCAM increases T cell activation while costimulation with CD318 inhibited activation of T cells (Supplemental Figure 3), suggesting that CD318 may have an inhibitory/modulatory role compared with ALCAM’s activating role on T cell activity.

The establishment of *CD6* expression in kidney-infiltrating T cells and ALCAM expression in kidney structural cells and antigen-presenting immune cells is supportive of a pathogenic role for the CD6/ALCAM pathway in the nephritic disease process.

### CD6 and ALCAM are overexpressed in murine models of systemic autoimmunity.

**Figure 3:**
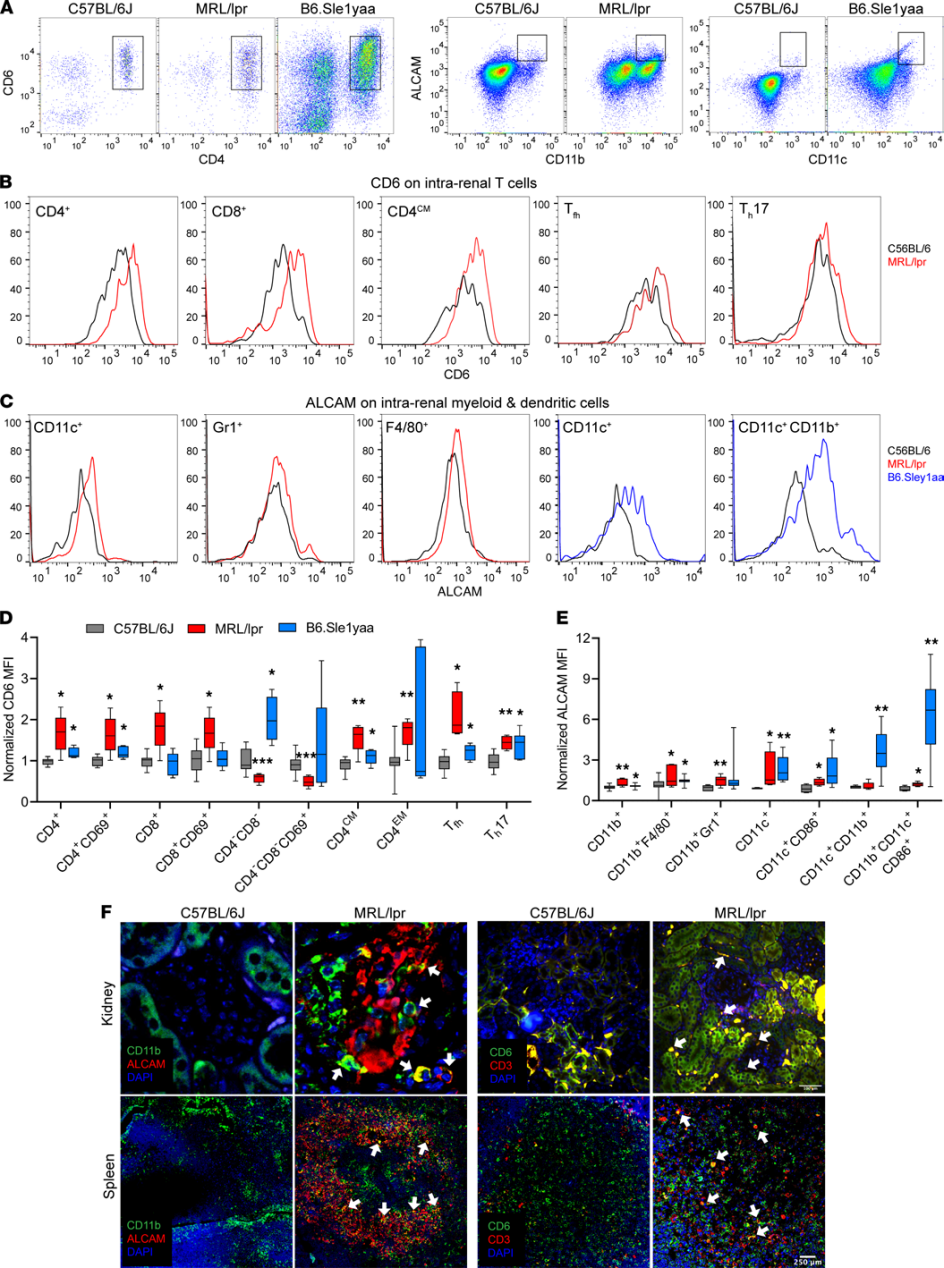
Characterization of ALCAM and CD6 expression on immune cells in murine models of SLE. Kidney cells from C57BL/6J (females, *n =* 8–14, 8–10 months old), MRL/lpr (females, *n =* 5–8, 8 months old) and B6.Sle1yaa (females, *n =* 7, 9–12 months old) were stained for CD6, ALCAM, and immune cell markers and then analyzed by flow cytometry to determine the CD166 and CD6 expression levels in various immune cell types. (**A**) Representative dot plots of CD4 versus CD6, pregated on CD3^+^ cells; CD11b versus ALCAM and CD11c versus ALCAM, pregated on live cells from C57BL/6J, MRL/lpr, and B6. Sle1yaa mice. (**B**) Representative histograms of C57BL/6J (black) and MRL/lpr (red) mice show CD6 expression on CD4^+^ and CD8^+^ T cells, CD4^+^ central memory T cells (CD62L^+^CD44^+^), CD4^+^ effector/effector memory T cells (CD62L^–^CD44^+^), CD4+ naive T cells (CD62L^+^CD44^–^), Tfh (CD45^+^CD4^+^CXCR5^+^PD1^+^) and Th17 cells (CD45^+^CD4^+^IL17a^+^). (**C**) Representative ALCAM histogram overlay for C57BL/6J (black), MRL/lpr (red) and B6. Sle1yaa (blue) mice showing granulocytes, macrophages, and CD11c^+^ and CD11c^+^/CD11b^+^ dendritic cells. (**D**) Normalized CD6 MFI of T cell subtypes were plotted for C57BL/6J (black) and MRL/lpr (red) and B6. Sle1yaa (blue). Data shown as standard box and whisker plots representing 25th, 50th, and 75th percentiles ± min/max. (**E**) CD166 MFI on myeloid and dendritic cells were normalized to C57BL/6J and plotted for C57BL/6J (black) and MRL/lpr (red) and B6. Sle1yaa (blue). (**F**) Kidney and spleen from MRL/lpr (left) or C57BL/6J (right) mice were stained for CD11b and CD166 or CD3 and CD6 and assessed by immunofluorescence microscopy. CD166 (red) expression was increased and colocalizes with CD11b^+^ (green) cells. CD6 (red) expression was increased in lupus mice kidney and colocalizes with CD3^+^ (green) cells. White arrow indicates colocalization. Images representative of 3–5 mice per group. Scale bar represents 100 mm for kidney and 250 mm for spleen. Comparisons between groups were done by Mann-Whitney *U* test. ***P < 0.001; **P < 0.01; *P < 0.05.

To determine if mouse models of SLE could be used to test hypotheses regarding our observations in humans, we next examined CD6 and ALCAM expression in MRL/MpJ-*lpr*/*lpr* (MRL/lpr) and B6.Sle1yaa strains of mice. Both of these strains develop spontaneous systemic autoimmunity that resembles that of human SLE, including hallmark characteristics such as B and T cell hyperactivity, autoantibodies, circulating immune complexes, complement consumption, and glomerulonephritis (37, 38). Using flow cytometry, we examined the expression of CD6 and ALCAM on T cells and monocytes, respectively, from kidneys of both MRL/lpr and B6.Sle1yaa mice at older than 6 months of age when systemic autoimmunity and renal disease are well-established, and compared them to C567BL/6, a strain of mice that does not develop spontaneous autoimmunity. Renal CD4^+^ T cells of MRL/lpr and B6.Sle1yaa both exhibited greater CD6 expression than the control strain as measured by mean fluorescence intensity (MFI) of CD6 staining. Interestingly, MRL/lpr mice exhibited a marked elevation in MFI of 68.5% while B6.Sle1yaa mice had a more modest increase of 19.4% over the control mice (Figure 3, A, B, and D). Compared with the kidneys from the C57BL/6 control, the percentage of intrarenal CD6^+^ CD3^+^ T cells was increased by about 2-fold, both in MRL/lpr (*P <* 0.0001) and B6.Sle1yaa mice (*P <* 0.0001). This increase was observed across CD4 subsets important in systemic autoimmunity/effector memory, central memory, T follicular helper cells (Tfh), and T helper 17 cells (Th17) (Figure 3, B and D). The MRL/lpr mice had higher CD6 MFI on CD8^+^ T cells with 81.2% greater expression over control mice, while the pattern was reversed on CD4^–^CD8^–^ T cells with B6.Sle1yaa mice expressing greater levels of CD6 (Figure 3D). The CD4^–^CD8^–^ T cells are predominantly described in mice due to aberrant expansion; however, there have been a few reports in human disease (39, 40). Within the renal monocyte population, dendritic cells (DCs) and, in particular, activated DCs (CD11c^+^CD11b^+^CD86^+^) exhibited the greatest increase in ALCAM compared with the C567BL/6 control strain (Figure 3, C and E). Compared with the kidneys from the C57BL/6 control, the percentage of intra-renal ALCAM^+^ CD11b^+^ myeloid cells was significantly increased both in MRL/lpr (*P <* 0.01) and B6.Sle1yaa mice (*P <* 0.01). T cells and monocytes isolated from spleens of the lupus mouse strains exhibited similar but smaller increases in both CD6 and ALCAM compared with C567BL/6 mice, suggesting that this pathway may be important both for systemic autoimmunity and end organ disease (Supplemental Figure 4).

When kidney tissue from MRL/lpr mice was immunofluorescently stained for ALCAM (Figure 3F), increased numbers of CD11b^+^ myeloid cells were seen in LN kidneys as expected (stained green), several of which coexpressed ALCAM (CD166, costained as yellow), although significant numbers of non-CD11b^+^ cells were also noted to be positive for ALCAM (stained red), the identity of which await elucidation. Similar findings were noted within the spleens of lupus mice, which exhibited enlarged marginal zones with rich cellular expression of ALCAM (CD166), some of which also coexpressed CD11b (costained yellow). When CD6 expression was examined, CD6 and CD3 coexpressing cells (stained yellow) were noted in both the kidneys and spleens of lupus mice, with these being more frequent than those seen in control C57BL/6 mice. Interestingly, the lupus spleens also exhibited cells that were discordant for CD3 and CD6 expression, stained green or red, respectively.

### CD6 blockade prolongs survival and alters renal pathology in mice with spontaneous LN.

Given the increased expression of CD6 and ALCAM in MRL/lpr mice, we evaluated the role of CD6/ALCAM in lupus by blocking the pathway using a mouse CD6-specific monoclonal antibody (anti-CD6). Starting at 9 to 10 weeks of age (close to the time when autoimmune disease is detected) and continuing up until 29 weeks of age (when systemic autoimmune disease is well-established), MRL/lpr mice were treated with anti-CD6, isotype control, cyclophosphamide, or mycophenolate mofetil (MMF). The latter 2 control groups of cyclophosphamide and MMF were used to provide mechanistic comparisons of drug approaches that are currently used to treat SLE and LN. Cyclophosphamide is a nonspecific immunosuppressant that cross-links DNA to prevent proliferation, while MMF widely targets both T and B cells. An additional nondiseased congenic control group consisting of mice from the parent strain MRL/MpJ, which do not develop disease until much later in life, was included as well.

**Figure 4:**
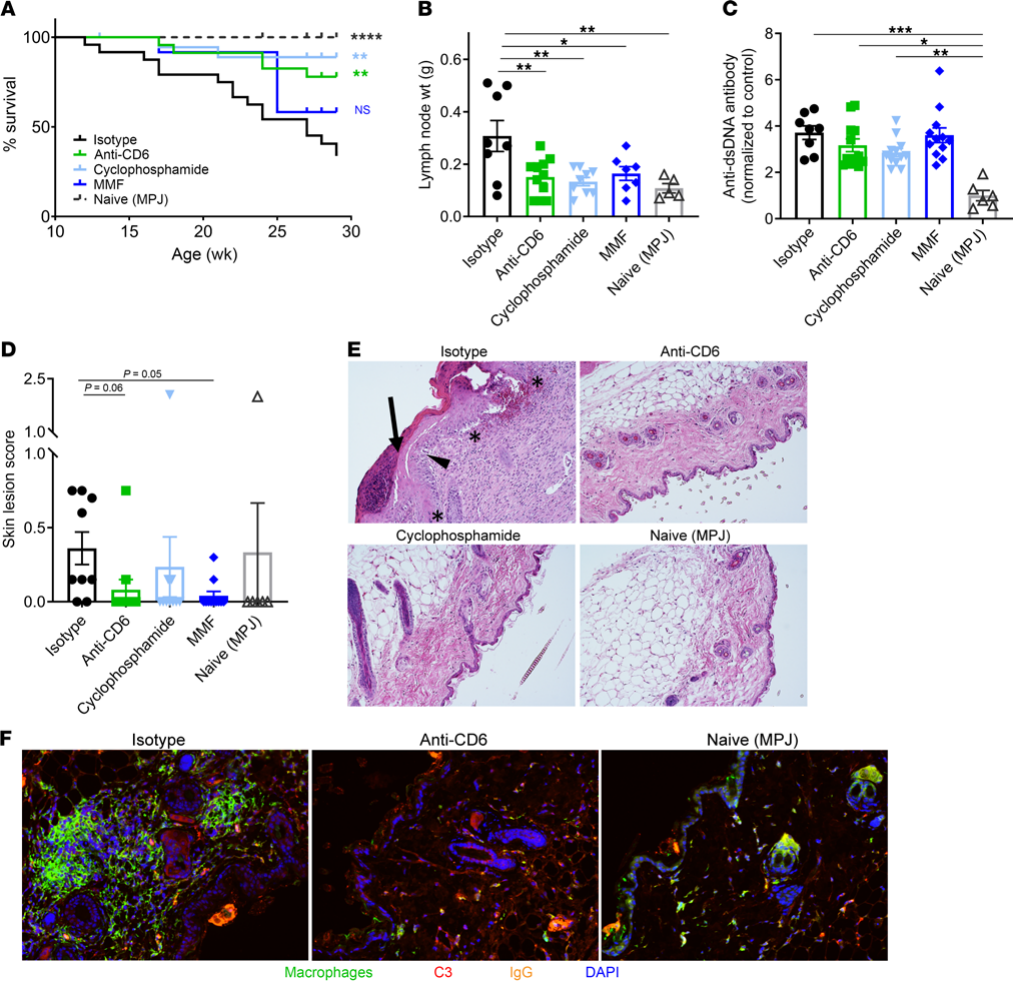
CD6 blockade improves survival and disease in MRL/lpr model of SLE. Female MRL/*lpr* mice at 9 to 10 weeks of age were treated with either anti-CD6 monoclonal antibody (60 μg/dose, i.p. twice per week, *n =* 12), isotype control (60 μg/dose, i.p. twice per week, *n =* 12), cyclophosphamide (25 mg/kg, once per week, *n =* 12), or mycophenolate mofetil (MMF; 50 mg/kg, oral gavage daily, *n =* 12). A group of MRL/MpJ mice (*n =* 6), a congenic control strain, were included in the study. (**A**) Kaplan-Meier curve depicting survival by treatment group (*n =* 10–12 mice per group). (**B**) Lymphadenopathy as assessed by average of the weight of the left and right inguinal lymph nodes at termination. (**C**) Serum levels of anti-dsDNA autoantibodies as measured by ELISA. (**D**) Scoring of macroscopic skin lesions at termination (29 weeks). (**E**) Skin histopathology of treated MRL/lpr and MPJ control mice. Arrow points to hyperkeratosis, asterisks indicate damage of the dermal-epidermal junction, and black triangle points to large cellular infiltrates into the dermis. (**F**) Skin sections stained for IBA1 (green) to identify macrophages, C3 (red) to delineate complement, IgG (orange) to identify immune complexes, and DAPI (blue) to identify cell nuclei. Data are representative of 2 independent experiments. Bar graphs present mean ± SE. Comparisons between groups were evaluated using 1-way ANOVA with multiple-comparisons test against the isotype group. ***P < 0.001; **P < 0.01; *P < 0.05 versus isotype.

SLE is characterized by B and T cell hyperactivity, autoantibodies, and involvement of multiple organs, including skin, in both murine and human disease. To first determine whether CD6 blockade could inhibit systemic autoimmunity, we examined extra-renal measures of disease in the MRL/lpr mice. At termination (29 weeks), CD6 blockade reduced lymphoid hyperplasia, suggesting significant inhibition of lymphocyte hyperproliferation (Figure 4B). Levels of anti-dsDNA antibodies were modestly reduced in anti-CD6 and cyclophosphamide-treated mice, although differences compared with the isotype control group were not significant (Figure 4C).

Patients with SLE commonly experience involvement of the skin, with development of cutaneous lesions. Similarly, MRL/lpr mice develop severe skin lesions as a manifestation of autoimmune disease. Macroscopic scoring of these lesions showed significant improvement in skin disease in anti-CD6–treated mice compared with the isotype control group (Figure 4D). Isotype control–treated mice displayed abnormal skin histopathology, including hyperkeratosis, damage to the dermal-epidermal junction, and large cellular infiltrates into the dermis (Figure 4E). Anti-CD6 treatment ameliorated these pathologies with reduced epidermal thickening, fewer cellular infiltrates, and an appearance more similar to healthy control sections from MPJ mice than to the isotype control–treated mice. Immunofluorescence staining for IBA1, a macrophage-specific calcium-binding protein, was decreased in the skin of anti-CD6–treated mice compared with the isotype group (Figure 4F). Thus, blockade of CD6 appears to dampen the recruitment and expansion of key leukocyte types (including T cells and macrophages) into involved skin in SLE.

**Figure 5:**
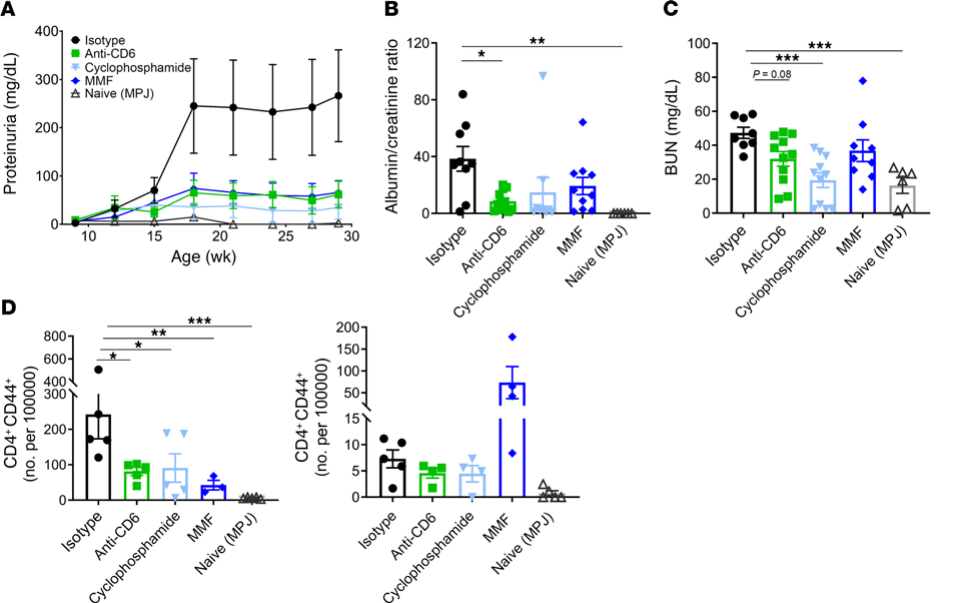
CD6 blockade improves renal function in MRL/lpr model of SLE. Female MRL/*lpr* mice at 9 to 10 weeks of age were treated with either anti-CD6 monoclonal antibody (60 μg/dose, i.p. twice per week, *n =* 12), isotype control (60 μg/dose, i.p. twice per week, *n =* 12), cyclophosphamide (25 mg/kg, once per week, *n =* 12), or mycophenolate mofetil (MMF; 50 mg/kg, oral gavage daily, *n =* 12). A group of MRL/MpJ mice (*n =* 6), a congenic control strain, were included in the study. (**A**) Longitudinal proteinuria as measured by uristix. (**B**) Terminal urine albumin/creatinine ratio and (**C**) BUN levels. (**D**) Detection of renal-infiltrating CD44^+^ CD4^+^ and CD8^+^ T cells by flow cytometry. Data are presented as mean ± SE and are representative of 2 independent experiments. Comparisons between groups were evaluated using 1-way ANOVA with multiple-comparisons test against the isotype group. ****P < 0.0001; ***P < 0.001; **P < 0.01; *P < 0.05 versus isotype.

**Figure 6:**
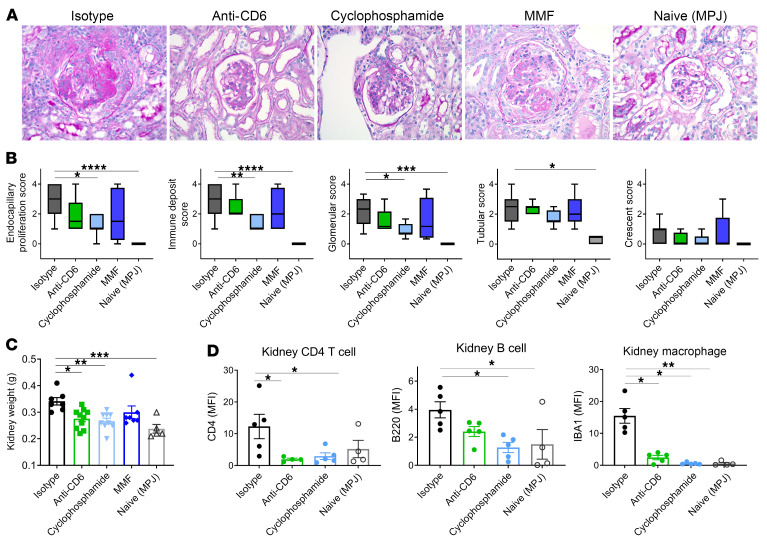
Anti-CD6 treatment inhibits renal pathology in the MRL/lpr model of SLE. Female MRL/*lpr* mice at 9 to 10 weeks of age were treated with either anti-CD6 monoclonal antibody (60 μg/dose, i.p. twice per week, *n =* 12), isotype control (60 μg/dose, i.p. twice per week, *n =* 12), cyclophosphamide (25 mg/kg, once per week, *n =* 12), or mycophenolate mofetil (MMF; 50 mg/kg, oral gavage daily, *n =* 12). A group of MRL/MpJ mice (*n =* 6), a congenic control strain, were included in the study. (**A**) Images of H&E sections from kidney of treated MRL/lpr and MPJ control mice. Magnification is 400×. (**B**) Pathology scores of H&E sections as scored by a blinded pathologist. Glomerular scores were assigned based on crescents, endocapillary hypercellularity, and immune material deposition while tubular scores were assigned based on casts/dilatation and interstitial inflammation. Scoring data are presented as box and whisker plots representing 25th, 50th, and 75th percentiles ± min/max. (**C**) Kidney weight at termination. Inflammation results in increased tissue weight. (**D**) Detection of CD4, B220, and IBA1 in immunofluorescently stained kidney sections. Data are presented as mean ± SE. All data represent 2 independent experiments. Comparisons between groups were evaluated using 1-way ANOVA with multiple-comparisons test against the isotype group. ***P < 0.001; **P < 0.01; *P < 0.05 versus isotype.

Treatment with anti-CD6 led to improved renal function in mice with spontaneous lupus. In the group that received anti-CD6, proteinuria remained consistently lower than the isotype control group, and was similar to the MMF- and cyclophosphamide-treated groups (Figure 5A). Both urine albumin/creatinine ratio and blood urea nitrogen (BUN) levels at termination were consistent with in-life proteinuria (Figure 5, B and C), suggesting that CD6 blockade inhibited loss of renal function. Albumin/creatinine ratio was not significant for the MMF and cyclophosphamide control groups, but this appeared to be due to one outlier in each group. Improvements in measures of renal function were supported by improvements in renal pathology. Histological examination of kidney tissue revealed decreasing trends in endocapillary proliferation, immune deposits, and glomerular damage in anti-CD6–treated mice, similar to MMF treatment (Figure 6, A and B). Furthermore, anti-CD6 mice demonstrated significantly lighter kidney weights, which is suggestive of less inflammation (Figure 6C).

In order to examine the mechanisms underlying the improved renal disease following anti-CD6 blockade in MRL/lpr lupus mice, we examined the cellular infiltrates. Anti-CD6 blockade was associated with significant reduction in infiltrating CD4^+^ T cells, including infiltrating effector/memory T cells (CD44^+^; Figure 5D). MMF-treated mice, while exhibiting a similar decrease in CD44^+^ CD4^+^ T cells, showed an increase in CD44^+^ CD8^+^ T cells, which was different compared with the other treatment groups. Examination of renal infiltration by immunofluorescence staining of kidney tissue for CD4^+^ T cells (CD4), B cells (B220), and macrophages (IBA1), demonstrated significant decreases in staining of CD4 cells and macrophages, and a nonsignificant decrease in B cell staining (Figure 6D). These differences were comparable to those changes observed in the MMF- and cyclophosphamide-treated groups (Figure 6D).

Approximately half of patients with SLE develop LN, which has a significant impact on mortality. Patients with LN also have a higher standardized mortality ratio and die earlier than patients with SLE without LN (41–43). MRL/lpr animals treated with anti-CD6 exhibited significantly improved survival compared with isotype-treated mice, similar to that observed in the cyclophosphamide-treated animals (Figure 4A). Comparatively, MMF-treated animals did not exhibit a significant increase in survival, which may be a reflection of the differing mechanisms. Together, these data suggest that the CD6/ALCAM pathway plays a role in both renal disease as well as systemic disease in SLE.

### CD6 blockade ameliorates acute immune complex–mediated glomerulonephritis.

**Figure 7:**
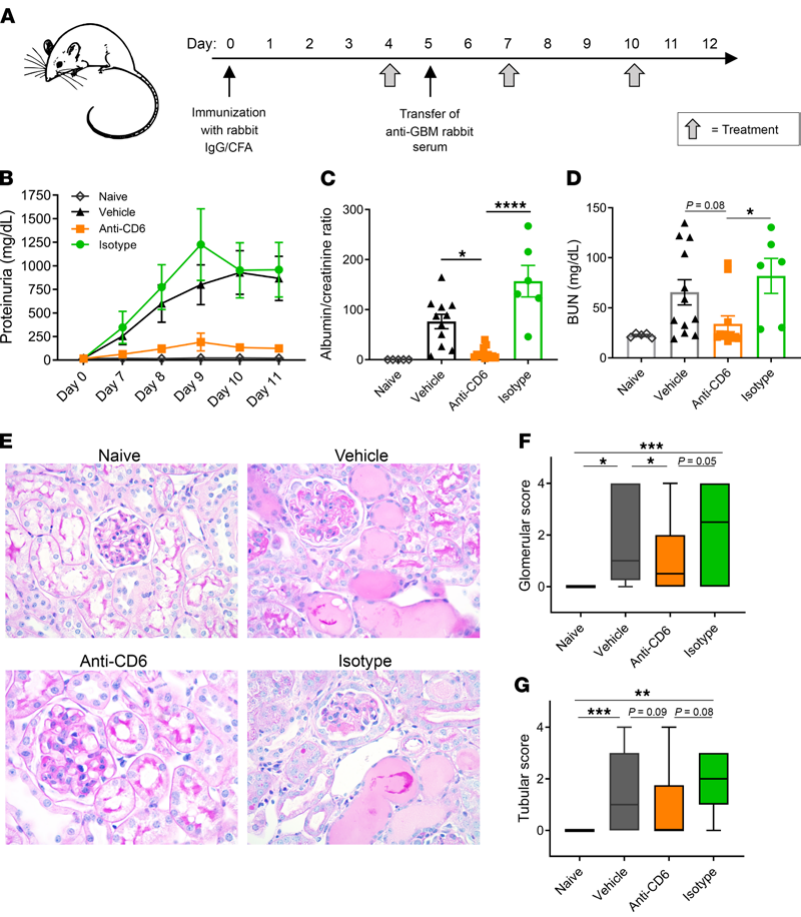
CD6 blockade inhibits immune complex–mediated renal damage. NTN was induced in female 129/SvJ mice at 10 weeks of age. Mice were immunized with rabbit IgG and CFA on day 0 to generate mouse anti–rabbit antibodies. At day 5, mice received nephrotoxic rabbit serum, which then cross-reacted with the mouse anti–rabbit antibodies, causing an antibody-mediated nephritis. Beginning day 4, mice were treated 3 times per week with anti-mouse CD6 (60 μg/dose; *n =* 12), vehicle control (*n =* 12), or isotype control (*n =* 6). Healthy mice (immunized with rabbit IgG, but not given nephrotoxic serum) were also included as a nondisease control (*n =* 5). (**A**) Schematic of the experimental design. (**B**) Longitudinal proteinuria as measured by uristix. Terminal urine albumin/creatinine ratio (**C**) and (**D**) serum BUN levels. Data are presented as mean ± SE. (**E**) Histological sections of renal tissue were scored blindly by a nephropathologist on a scale of 0–4. (**F**) Glomerular sections were assessed by scoring endocapillary proliferation, crescents, and immune deposits. (**G**) Tubular scores were determined by scoring tubular casts and interstitial inflammation. Scoring data are presented as box and whisker plots depicting 25th, 50th, and 75th percentiles ± min/max. All data are representative of 2 independent experiments. Comparisons between groups were evaluated using 1-way ANOVA with multiple-comparisons test. ****P < 0.0001; ***P < 0.001; **P < 0.01; *P < 0.05.

To specifically examine the contribution of CD6 to lupus nephritis, we utilized the mouse model of nephrotoxic serum nephritis (NTN). The NTN model is an induced model of acute immune complex–mediated glomerulonephritis that is used to study renal-specific events in lupus nephritis; the acute model accurately reflects the role of various molecular mediators in LN within a short time frame (44). To better evaluate whether the CD6/ALCAM pathway could be involved in the pathology of LN, we blocked the pathway in this model using anti-CD6. To induce disease, mice were immunized against rabbit IgG at day 0 to induce formation of anti–rabbit IgG antibodies (Figure 7A). Then at day 5, polyclonal rabbit antibodies raised against mouse glomerular basement membrane proteins were injected into the mice, resulting in immune-complex formation in the kidney followed by inflammation. Beginning at day 4, mice were treated with vehicle (PBS), isotype control, or anti-CD6; treatments were not administered earlier to minimize interference with disease induction. Additionally, a disease-naive group was included that was immunized against rabbit IgG but did not receive rabbit antibodies. Starting at day 8, mice treated with anti-CD6 maintained significantly lower levels of proteinuria over the length of the study compared with control mice (*P <* 0.001; Figure 7B). In control mice, proteinuria increased daily from day 7 and peaked at day 9 in mice that received isotype control and at day 10 in mice that received the vehicle control (*P <* 0.001; Figure 7B). As a more accurate measure of kidney function, albumin and creatinine in urine were quantified at termination to determine the albumin/creatinine ratio (Figure 7C). The ratio was significantly lower in anti-CD6–treated mice, only slightly elevated over disease-naive mice, suggesting that CD6 blockade protected kidney function. Furthermore, BUN, a second measure of kidney function in serum, was significantly decreased in this group (*P <* 0.01; Figure 7D). The observed improvements in disease measures were not due to weaker disease induction in these mice, as the levels of mouse anti–rabbit IgG and rabbit anti–mouse glomerular basement membrane antibodies were similar across all groups (Supplemental Figure 5). The improvement in kidney function was also accompanied by improvements in renal pathology, with a significant decrease in glomerular pathology and a decreasing trend in tubular pathology (Figure 7, E–G).

**Figure 8:**
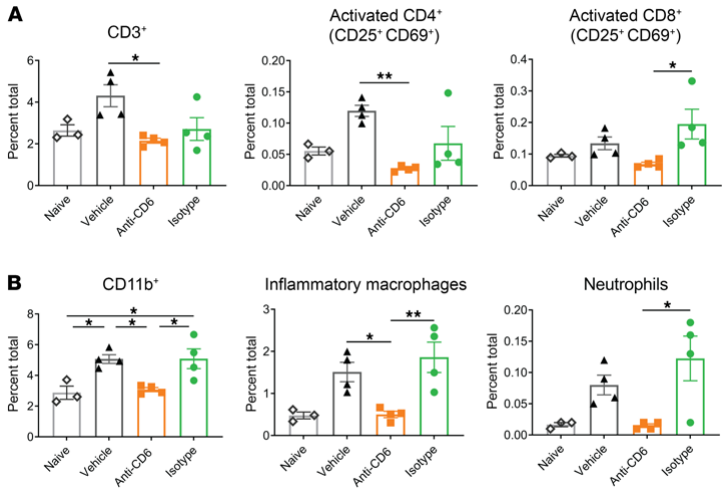
Anti-CD6–treated NTN mice exhibit decreased renal infiltration of inflammatory immune cells. NTN was induced in female 129/SvJ mice at 10 weeks of age. Mice were immunized with rabbit IgG and CFA on day 0 to generate mouse anti–rabbit antibodies. At day 5, mice received nephrotoxic rabbit serum, which then cross-reacted with the mouse anti–rabbit antibodies, causing an antibody-mediated nephritis. Beginning day 4, mice were treated 3 times per week with anti–mouse CD6 (60 μg/dose; *n =* 12), vehicle control (*n =* 12), or isotype control (*n =* 5). Healthy mice (immunized with rabbit IgG, but not given nephrotoxic serum) were also included as a nondisease control (*n =* 6). At day 11 to 12, mice were sacrificed and kidneys were harvested to examine the prevalence of immune cells by flow cytometry. (**A**) Prevalence of CD3^+^ and activated (CD25^+^ CD69^+^) CD4^+^ and CD8^+^ T cells in renal tissue. (**B**) Prevalence of CD11b^+^ monocytes, inflammatory macrophages (CD11b^+^F4/80^lo^Ly6C^hi^), and neutrophils (CD11b^+^GR1^hi^). Data are presented as mean ± SE and represent 2 independent experiments. Comparisons between groups were evaluated using 1-way ANOVA with multiple-comparisons test. ****P < 0.0001; ***P < 0.001; **P < 0.01; *P < 0.05.

During the course of glomerulonephritis in this model, inflammatory immune cells such as Teff, inflammatory macrophages, and neutrophils infiltrate the kidney to participate in and drive the disease process. In anti-CD6–treated mice, there were fewer total lymphocytes (CD45^+^) detected in the kidney. Total T cells (CD3^+^) and CD4^+^ and CD8^+^ T cell numbers were diminished in the kidneys of anti-CD6–treated mice. More importantly, the prevalence of T cells with an activated phenotype (CD25^+^ CD69^+^) were significantly decreased compared with the isotype control (Figure 8A). Although anti-CD6 only targets T cells, the prevalence of CD11b^+^ myeloid cells, inflammatory macrophages, and neutrophils also exhibited significant decreases with CD6 blockade (Figure 8B), suggesting that targeting of the CD6 on T cells led to a less inflammatory environment with a subsequent decrease in downstream immune cell recruitment.

**Figure 9:**
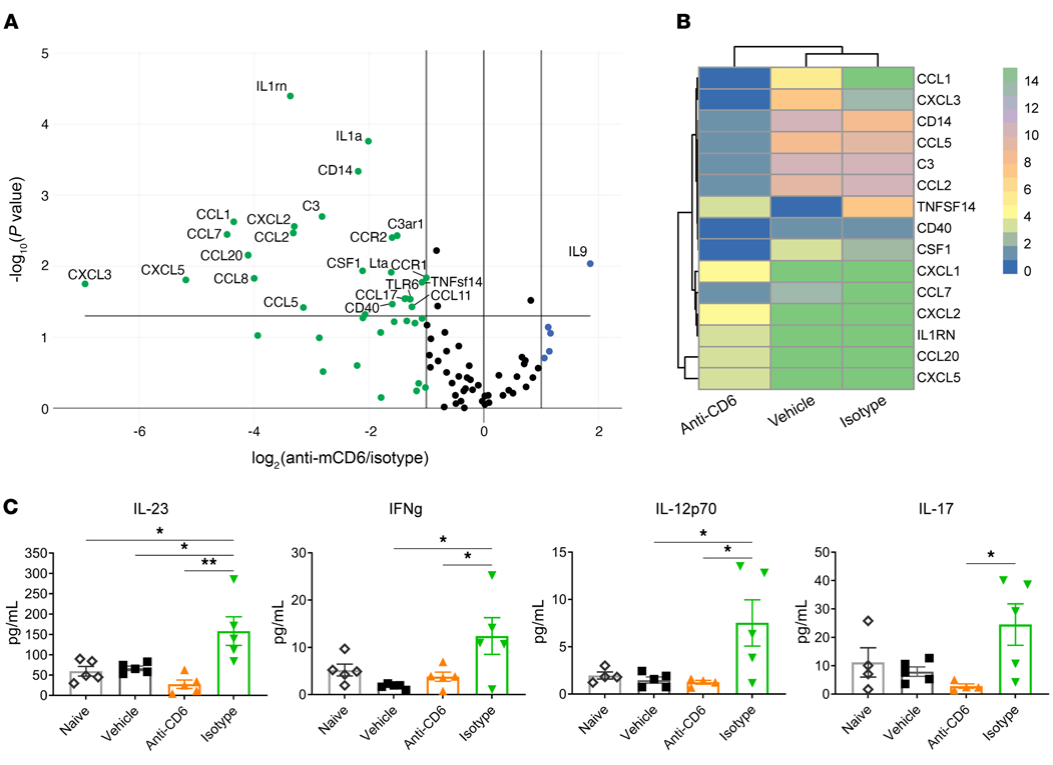
Blockade of CD6 decreases expression of inflammatory cytokines/chemokines. NTN was induced in female 129/SvJ mice at 10 weeks of age. Mice were immunized with rabbit IgG and CFA on day 0 to generate mouse anti–rabbit antibodies. At day 5, mice received nephrotoxic rabbit serum, which then cross-reacted with the mouse anti–rabbit antibodies, causing an antibody-mediated nephritis. Beginning day 4, mice were treated 3 times per week with anti–mouse CD6 (60 μg/dose; *n =* 12), vehicle control (*n =* 12), or isotype control (*n =* 5). Healthy mice (immunized with rabbit IgG, but not given nephrotoxic serum) were also included as a nondisease control (*n =* 6). At day 11 to 12, mice were sacrificed and kidneys were harvested to analyze RNA and protein levels of inflammatory markers. (**A**) Volcano plot of results of PCR array examining expression of 86 genes associated with inflammation in RNA isolated from kidneys of anti-CD6– and isotype-treated mice. (**B**) Heat map of genes that differed by more than 3-fold between isotype- and anti-CD6–treated mice. (**C**) Protein levels of select genes (IL-23, IFN-γ, IL-12p70, and IL-17) in renal tissue, as quantitated by flow-based ELISA. Data represent mean ± SE. Comparisons between groups were evaluated using 1-way ANOVA with multiple-comparisons test. **P < 0.01; *P < 0.05.

To better examine the inflammatory milieu in the kidney, we performed a gene expression analysis to quantitate levels of chemokines, cytokines, and receptors associated with the induced inflammation. Comparison of anti-CD6–treated versus isotype-treated mice revealed decreases in a number of factors including C3 complement, CXCL3, and CCL2/MCP-1 (Figure 9, A and B). A more than 2-fold increase in expression was only detected for IL-9, a cytokine that has been reported to enhance Treg function (45). In addition to RNA levels, we also measured protein levels of a panel of cytokines in renal tissue. IL-12p70, IL-17, IL-23, and IFN-γ were significantly lower in anti-CD6–treated mice versus isotype-treated mice (Figure 9C). Vehicle-treated mice had low levels equivalent to naive mice. While the inflammatory cytokine levels in vehicle-treated mice were lower than expected, inflammation was present as indicated by disease and renal immune cell infiltration (Figure 7 and Figure 8). Together, these data indicate that the CD6/ALCAM pathway is involved in this induced acute glomerulonephritis model at the level of the kidney.

## Discussion

T cells are thought to be instrumental in the development and progression of SLE and LN (46–48). Patients with SLE exhibit aberrant T cell signaling, altered gene expression profiles, increased Th17 responses (49), and impaired Treg function (49, 50), while depletion of T cells mitigates development of nephritis in MRL/lpr mice (51, 52). Although T cells are implicated in the pathogenesis of SLE, specific targeting of pathogenic T cells without global immunosuppression or suppression of regulatory T cell subsets has proven challenging. Current standard of care utilizes more broadly immunosuppressive drugs, such as MMF and cyclophosphamide, to suppress T cell responses, often with significant toxic side effects. Thus, more targeted therapies are needed to effectively treat SLE and LN. CD6 is expressed on Teff cells, but not Tregs, and the CD6/ALCAM pathway is involved in T cell–associated immune processes, including T cell activation and migration, and consequently provides an ideal target. Hence, here we elucidate a role for the CD6/ALCAM pathway in SLE/LN and demonstrate its potential as a highly specific therapeutic target.

Previously, we had observed high levels of uALCAM in patients with LN. We reported that uALCAM levels could distinguish patients with SLE with active renal involvement from patients with quiescent or no prior nephritis, and predict long-term renal deterioration (33, 34). Here, by analyzing uALCAM data from an extended cross-sectional cohort of patients of different ethnicities, we were able to validate uALCAM in a diverse set of patients as a biomarker that is able to discern active renal involvement in SLE versus inactive or no renal involvement. Notably, in all ethnicities, the uALCAM level correlated with rSLEDAI, a clinical measure of LN disease severity (Figure 1, E–H), linking this protein more closely with renal disease progression.

Given that uALCAM is relatively low in patients with nonrenal lupus who were systemically active, our results suggest uALCAM reflects renal rather than systemic activity. Indeed, we have assayed serum and urine ALCAM in the same cohort of subjects (data not shown), where we find that uALCAM was able to significantly discriminate active LN from other patients with SLE (AUC values > 92%), whereas sALCAM failed to do so. Moreover, the relative superiority of uALCAM over some current urinary or plasma markers has been previously validated (34), although this needs to be confirmed in additional studies. Interestingly, in diabetic nephropathy, serum concentrations of ALCAM have been shown to be elevated and inversely correlated with renal function, while ALCAM expression was upregulated both in glomeruli and tubules, mainly in podocytes (53), though uALCAM was not examined. Clearly, a more comprehensive survey of renal diseases is warranted in order to establish the specificity of this urinary biomarker.

ALCAM is expressed on a variety of cell types, including nonhematopoietic cells and APCs, and can be further upregulated under cell-activating or proinflammatory conditions (27, 54). Immunologically, ALCAM binds to CD6, which is mainly expressed on Teff cells to regulate immune processes such as T cell activation, differentiation, and migration (55). Therefore, to identify the specific cells that expressed CD6 and ALCAM in LN, we examined single-cell RNA-Seq data generated from renal biopsies of patients with LN as part of the Accelerated Medicines Partnership (35). We found *CD6* and *ALCAM* expression within key cell populations. CD6 expression was established in renal-infiltrating T cells that contribute to the pathology of lupus nephritis, while high ALCAM expression was established in structural renal cells that are subject to damage during LN disease. Given that urine would capture proteins released from the inflamed kidney, it is likely that the high uALCAM levels observed during active LN are derived from these high ALCAM-expressing structural cells. Cells damaged by inflammation may shed or release ALCAM, or alternatively, higher levels of cell-surface ALCAM induced by inflammatory cytokines can be cleaved by the cell membrane metalloprotease ADAM-17 (56), thereby releasing the protein into the urine. *ALCAM* was also highly expressed on infiltrating APCs in the kidney. This provides not only another possible source of uALCAM but importantly highlights potential immunologically significant interactions between CD6 and ALCAM-expressing cells in the kidney that could further exacerbate disease. Interactions via CD6 and ALCAM include: (a) interaction of CD6^+^ T cells with ALCAM^+^ APCs resulting in restimulation and maintenance of pathogenic T cell responses in the kidney; (b) interaction of CD6^+^ T cells with ALCAM^+^ kidney structural cells, which are also known to express MHCI and/or MHCII and would also result in restimulation of T cells; or (c) CD6^+^ T cells interacting with ALCAM on renal structural cells to aide in further infiltration into the inflamed tissue. Concordance of these interactions may aid in sustaining high levels of pathogenic T cell activity and trafficking, including release of proinflammatory cytokines, recruitment of inflammatory cells, and increased destruction of renal tissue, all of which further perpetuate the inflammatory cycle. Future mechanistic studies will help further clarify these (and perhaps other) possible effects of CD6/ALCAM interactions in LN, and determine their relative contributions.

While these studies focus on CD6/ALCAM interactions, CD6 and CD318 interactions have not been ruled out as contributing to disease. However, we found that levels of CD318 in serum and urine did not distinguish between lupus disease states, and little expression was detected within renal cells, suggesting that this ligand is not a large driver of the renal pathology in LN. Furthermore, costimulation with CD318 inhibited T cell activation, an observation in line with the recent paper by Ruth et al. (57) in which blockade of CD6 was shown to enhance killing of tumor cells by CD8^+^ T cells and NK cells.

The significant correlation between ALCAM and disease status and the expression of CD6 and ALCAM on renal cell types involved in LN led us to hypothesize that the CD6/ALCAM pathway is a driver of disease. To confirm the role of the CD6/ALCAM pathway in SLE pathogenesis, we tested this pathway in 2 different disease models using an anti-CD6 antibody. When we inhibited the CD6/ALCAM pathway in the MRL/lpr strain and the NTN model, this resulted in decreased numbers of kidney-infiltrating CD4 and CD8 T cells, including activated and effector/memory subsets. Even though an anti-CD6 antibody specifically targets T cells, both flow cytometry and RT-qPCR analyses demonstrated a wider effect beyond this population. The inflammatory environment was decreased in terms of both non–T cell chemokines/cytokines and kidney-infiltrating immune cells. Specifically, inhibition of the T cell response reduced the recruitment of myeloid cells, inflammatory macrophages, and neutrophils, cell types that exhibit aberrations in patients with SLE and also play a crucial part in disease. Neutrophils accumulate in the kidneys of patients, while renal infiltrating macrophages are associated with LN severity, renal damage, and poor clinical outcome (58, 59). Although T cells do not always act upstream of these innate cells, here T cells are a driving factor to activate and recruit other immune subsets. Consequently, our data suggest that blockade of CD6 on T cells can alter the trajectory of disease by not only inhibiting the T cell responses but also by indirectly reducing the involvement of other immune cells that contribute to disease pathogenesis.

Histologically, improvements in the renal pathology of anti-CD6–treated MRL/lpr mice did not reach statistical significance, though decreases were observed in scores for endocapillary hypercellularity, glomerular injury, and crescent formation (Figure 6B). However, MRL/lpr mice treated with MMF also did not exhibit significant improvements in pathology. It is possible that significant differences in pathology were not observed because tissue damage occurred very early on and could not be resolved within the time frame of the study, as the mechanisms of MMF and CD6 blockade may act downstream of events, such as immune deposition, that initiate early renal injury (Figure 6B). This is supported by the NTN model in which treatment was applied after the ability to create immune complexes was formed. Importantly, anti-CD6 treatment prevented functionally significant damage, as treated mice had significantly better renal function compared with isotype control mice as demonstrated by decreased albumin/creatinine ratios and BUN levels (Figure 5). Furthermore, the use of CD6 blockade that only targets T cells had an equivalent effect to MMF and cyclophosphamide, both potent immunosuppressors that target multiple immune cell populations.

**Figure 10:**
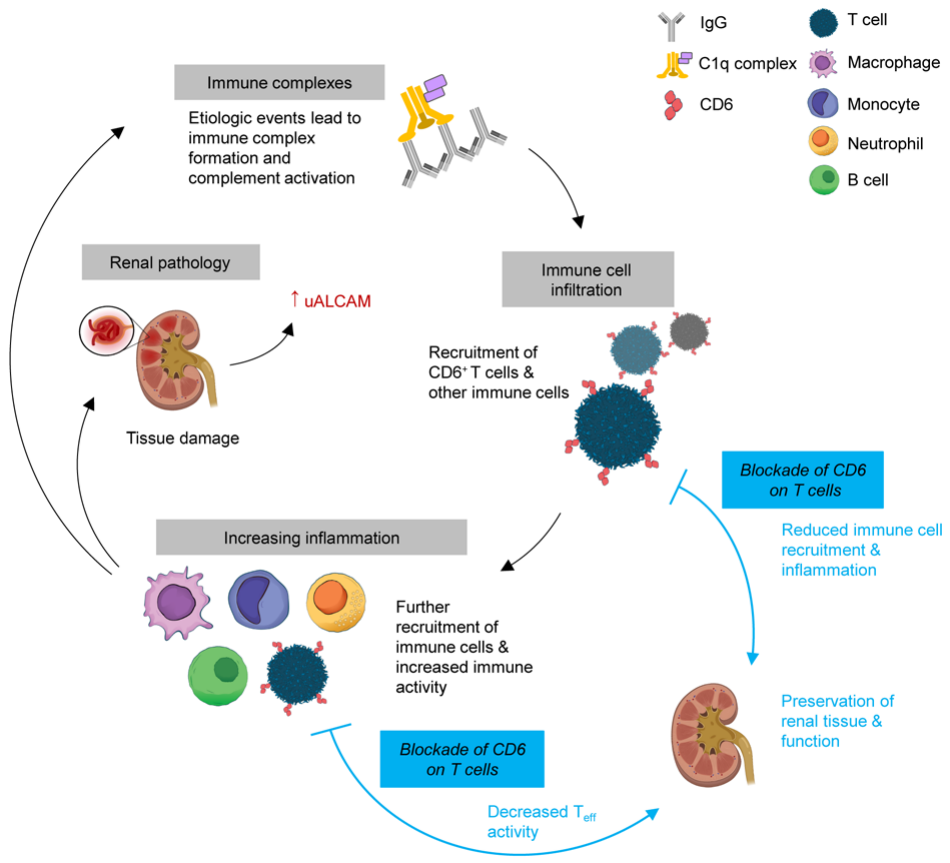
Schematic of CD6/ALCAM blockade in immune complex–mediated glomerulonephritis. Lupus autoantibodies bind to resident renal cells leading to immune-complex formation in the kidney and, subsequently, complement activation. Initial renal injury leads to recruitment of immune cells to the kidney including CD6^+^ T cells. The T cells release inflammatory cytokines that induce further recruitment of immune cells, including more T cells, monocytes, inflammatory macrophages, neutrophils, and B cells. Increased inflammation results in tissue damage, pathology, and release of ALCAM into the urine. The continued inflammation further exposes renal tissue to autoreactive antibodies, which continues the inflammatory cycle. Blockade of CD6 on T cells inhibits activation of T cells and their function, resulting in reduced inflammatory cytokines and chemokines, and thereby, reductions in immune cell recruitment.

Although mouse models cannot perfectly replicate disease in humans, the consistency of disease improvement across 2 different murine models that represent the disease events in LN advocates the involvement of this pathway in driving disease pathogenesis. Most importantly, CD6 blockade increased survival in MRL/lpr mice via a more targeted and specific mechanism than those affected by cyclophosphamide and MMF, which are both used as standard of care in the treatment of LN. Future work will further illuminate the downstream mechanistic consequences of CD6 blockade. However, its success in improving survival suggests that CD6 is involved in driving wider, systemic disease and supports the idea that a more targeted, less immunosuppressive drug mechanism can be successful in treatment. Based on the murine models, CD6 blockade does not prevent formation of early immune complexes but rather acts downstream to prevent later damage and inflammation due to immune cell infiltration and inflammatory cytokines/chemokines (Figure 10). Continued work will better assess the therapeutic potential of CD6 blockade in both mouse models (e.g., starting treatment after the onset of disease) and human subjects to determine whether inhibiting this pathway is a viable strategy for the treatment of LN and/or SLE. Currently, an anti-CD6 antibody (itolizumab) is being tested in the clinic for the treatment of LN (ClinicalTrials.gov, NCT04128579).

In summary, we conclusively demonstrate here that the CD6/ALCAM pathway is important in the pathogenesis of both SLE and LN. Not only are soluble levels of uALCAM, and possibly CD6, a strong biomarker of disease, but therapeutic targeting of this pathway may provide an effective treatment for multiple pathologies of SLE, including skin and renal manifestations.

## Methods

### Human studies

Methods pertaining to the human cohorts (60–61), quantification of ALCAM, IFN-γ, and TNF-α, assessment of CD318, and RNA-Seq data analysis (62), are found in the Supplemental Methods.

### Animal studies

All mice were purchased from Jackson Laboratories and bred and housed in pathogen-free facilities.

#### NTN model.

Nephrotoxic serum nephritis (NTN) was induced in female 129/SvJ mice at 10 weeks of age as previously described (63, 64). Briefly, mice were immunized with rabbit IgG and CFA on day 0 to generate mouse anti–rabbit antibodies. At day 5, mice received nephrotoxic rabbit serum, which then cross-reacted with the mouse anti–rabbit antibodies, causing an antibody-mediated nephritis similar in pathology to LN. In 2 independent experiments, mice were treated 3 times per week with an anti-CD6 monoclonal antibody (10D12; 60 μg/dose, *n =* 11 in first study, *n =* 12 in second study), vehicle control (*n =* 11 in first study, *n =* 12 in second study), or isotype control (60 μg/dose, *n =* 6 per study) by i.p. injection. Healthy mice (immunized with rabbit IgG, but not given nephrotoxic serum) were also included as a nondisease control (*n =* 7 in first study, *n =* 5 second study). The progress of kidney disease was monitored starting at day 7 via proteinuria as detected by the colorimetric dipstick assay. At study termination, spleen and kidney tissue were collected and processed into single-cell suspension to assess T cell and monocyte populations or snap frozen to test tissue levels of cytokine transcript and protein.

#### Animal models of spontaneous LN.

Two different animal models of spontaneous LN, both aged 6 months, were examined for the expression profiles of CD6 and ALCAM, including the MRL.*Faslpr* (MRL/lpr) model and the B6.Sle1yaa, a model on the C57BL/6 background that bears 2 genetic susceptibility loci for lupus, *Sle1* and *Yaa* (38). Spleen and kidney from these 2 lupus strains as well the C57Bl/6 healthy control strain were collected and either processed for FACS staining or snap-frozen in OCT freezing medium for sectioning and staining.

To assess the effects of CD6 blockade, female MRL/lpr mice were aged to 9 to 10 weeks of age before initiating treatment. In the first experiment, mice were treated with either anti-CD6 monoclonal antibody (60 μg/dose, i.p. twice per week; *n =* 12), isotype control (60 μg/dose, i.p. twice per week; *n =* 12), or cyclophosphamide (25 mg/kg, once per week; *n =* 8); MRL/MpJ mice (*n =* 10), a congenic strain, served as a healthy control. The second experiment included mice treated with either anti-CD6 monoclonal antibody (60 μg/dose, i.p. twice per week, *n =* 12), isotype control (60 μg/dose, i.p. twice per week; *n =* 12), cyclophosphamide (25 mg/kg, once per week; *n =* 12), or mycophenolate mofetil (MMF; 50 mg/kg, oral gavage daily; *n =* 12), and MRL/MpJ congenic controls (*n =* 6).

Mice were monitored weekly for weight changes and proteinuria, and scored for lymph node swelling and macroscopic skin lesions. The study was terminated when proteinuria exceeded 300 mg/dL in more than 50% of the isotype control group as determined by colorimetric dipstick assay (Albustix; Bayer). At termination, serum and urine were collected to assess serum cytokines, anti–DNA IgG (as described in ref. 65), BUN levels, and albumin/creatinine ratio. Quantification of BUN was performed using the QuantiChrom Urea Assay Kit (BioAssay Systems), albumin using the mouse albumin ELISA kit (Bethyl Laboratories), and creatinine using the QuantiChrom Creatinine Assay Kit (BioAssay Systems). All kits were used according to manufacturer’s instructions. Spleen and kidney tissue were collected and processed into single-cell suspension to assess T cell and monocyte populations, snap frozen to test tissue levels of cytokine transcript and protein, or fixed and paraffin-embedded to assess tissue pathology. Two independent experiments were performed.

#### Cell isolation and flow cytometry.

For assessment of CD6 and ALCAM expression in kidney and spleen of spontaneous models of LN, mice were perfused at sacrifice with ice-cold PBS, and spleens and kidneys were harvested. Tissue was mechanically disrupted, washed in cold PBS, and the pellet was suspended in DMEM with 1 mg/mL collagenase IV (Life Technologies) followed by 40 minutes of incubation in a 37°C shaker. Cells were further mechanically disrupted by passage through a 20-gauge syringe, incubated for 3 minutes with RBC lysis buffer (Sigma-Aldrich), and then single-cell suspensions were prepared for flow cytometry. Flow cytometry was performed as previously described (66, 67). Briefly, cells were blocked for 15 minutes with staining buffer (PBS, 3% BSA, 0.05% azide) followed by staining with fluorescently conjugated antibodies against markers of interest for 30 minutes. For live cell discrimination, cells were stained with Zombie aqua dye (BioLegend). The following dye- or biotin-coupled antibodies were purchased from BD Biosciences: CD45-APCCy7 (clone 30-F11), CD11b-FITC (clone M1/70), Gr1-BV421 (clone RB6-8C5), F4/80-PE (clone T45-2342), CD86-BV421 (clone GL1), CD11c-PECy7 (clone HL3), Ly6C-PerCPCy5.5 (clone AL-21), CD3-FITC (clone 145-2C11), CD4-APC (clone RM4-5), CD8-PECy7 (clone 53-6.7), CD69-BV421 (clone H1.2F3), CD44-FITC (clone IM7), CD62L-PECy7 (clone Mel-14), PDL1-BV421 (clone B7-H1), CXCR5-PECy7 (clone 2G8), CD25-APC (clone PC61), and IL17A-APCCy7 (clone TC11-18H10). CD166-APC (clone eBioALC48) and CD6-PE (clone IM348) were purchased from Invitrogen. Cell staining was analyzed using BD FACS Aria II (BD Biosciences). For kidney samples, at least 1 × 10^5^ cells were acquired on the leukocyte gate, as defined by CD45 positivity and size. For spleen, at least 5 × 10^4^ cells were acquired on the live cell gate, as defined by scatter properties and live cell dye staining. Data were analyzed using FlowJo version 10.

For CD6 blockade experiments, mice were perfused at sacrifice with ice-cold PBS, and spleens and kidneys were harvested and stored over night at 4°C in MACS Tissue Storage Solution (Miltenyi Biotec). The next day, kidneys were sliced into smaller pieces with a razor blade and then digested in 2 mg/mL collagenase (Worthington) for 30 minutes at 37°C. During the digestion process, samples were shaken every 10 minutes. Kidneys were then serially pipetted through progressively smaller pipette tips to achieve a single-cell suspension. Spleens were mashed through a 70 μm filter to achieve a single-cell suspension. Both tissues were subjected to a 15-minute RBC lysis on ice, filtered through an additional 70 μm filter, and then blocked for 30 minutes on ice with Fc Block (anti-CD16/CD32, BD Pharmingen) diluted 1:200 in 3% FBS in PBS. Kidney cells were then stained on ice for 30 minutes, and spleen cells were used as single-color controls. A total of 3 staining panels were designed to fully phenotype the infiltrates of immune cells in the kidney. Panel 1 focused on resident and inflammatory macrophages, myeloid DCs, DCs with Ly6G-FITC, Ly6C-PE, CD11b-AF700 and CD11c-Pacific Blue. Panel 2 focused on neutrophils and B cells with CD80-FITC, CD86-Pacific Blue, B220-PE, CD19-APCCy7, and MHCII-APC. Panel 3 focused on T cells and T cell activation with CD45–Alexa Fluor 700, CD3-PerCP, CD8-PeCy7, CD4-APC, CD44–Pacific Blue, CD25-FITC, and CD69-PE. Stained samples were then fixed with 2% PFA, stored overnight, and analyzed the next day.

#### Immunofluorescence.

Paraffin sections (5 μm) of kidney or spleen were deparaffinized and rehydrated, followed by antigen retrieval in citrate buffer for 5 minutes at a temperature of greater than 90°C.

For staining of CD3, CD6, CD11b, and ALCAM (CD166), slides were blocked with 5% normal rat serum in PBS for 30 minutes. The tissue was stained with anti-CD3 (CD3-12), anti-CD11b (EPR1344), anti-CD166 (B-6), and anti-CD6 (ab231314) at 4°C overnight. Sections were then washed and incubated with goat anti–rabbit IgG FITC/Cy3 and goat anti–mouse FITC/Cy3 (Jackson ImmunoResearch Laboratory) for 1 hour at room temperature. Sections were washed, mounted with Vectashield with DAPI (Vector Laboratories), visualized and imaged using a Nikon confocal microscope, and analyzed using NIS-Elements imaging software.

For staining of IBA1, C3, and mouse IgG, slides were blocked for 1 hour at room temperature with 20% horse serum and 0.05% triton in PBS. Slides were then incubated with primary antibody overnight at 4°C (stain 1: 1:250 rabbit anti-mouse IBA1, 1:100 goat anti-mouse C3, 1:500 donkey anti-mouse IgG AF647; stain 2: 1:100 rat anti-mouse B220 and 1:100 rabbit anti-mouse CD3). The following day, the slides were allowed to come to room temperature, washed and then incubated with fluorescence conjugated secondary antibodies for 1 hour (stain 1: 1:250 donkey anti-rabbit AF488, 1:100 donkey anti-goat AF594, and 1:500 donkey anti-mouse AF647; stain 2: 1:100 donkey anti-rat AF594 and 1: 100 donkey anti-rabbit AF488). Slides were washed, stained with DAPI, and mounted using Fluoromount-G. Sections were imaged using EVOS FL Auto 2 (Invitrogen) and quantified using ImageJ, using either cell counts or mean fluorescence intensity, as indicated.

#### Histology.

Kidney sections were deparaffinized and stained with hematoxylin and eosin (H&E) and periodic acid Schiff (PAS) by the Albert Einstein College of Medicine Histology and Comparative Pathology Core. Kidney sections were then analyzed and scored by a nephropathologist who was blinded to the treatment groups, as previously described (63). Glomerular scores were assigned based on the presence of crescents, endocapillary hypercellularity, and immune deposits. Tubular scores were assigned based on casts/dilatation and interstitial inflammation. Each category was assigned a score of 0 to 4, where 4 is severe disease and 0 is no disease or normal-appearing histology. Scores for the glomerular parameters (crescents, endocapillary proliferation, and immune deposits) were averaged to obtain a score for glomerular histology; scores for interstitial inflammation and tubular casts and dilatation were averaged to obtain a score for tubular histology.

#### PCR array.

RNA was isolated from frozen kidney tissue by homogenizing the tissue in TRIzol (ThermoFisher) and applying Qiagen’s RNeasy Mini kit (Qiagen) per the manufacturer’s instructions. RNA concentration and purity were determined using UV spectrophotometry, and cDNA was synthesized using Qiagen’s RT^2^ First Strand Kit (Qiagen) according to the manufacturer’s instructions. Subsequently, the cDNA was used in the RT^2^ Profiler PCR Array Mouse Inflammatory Response & Autoimmunity (Qiagen) to assess expression of 84 genes associated with immune inflammation (https://geneglobe.qiagen.com/us/product-groups/rt2-profiler-pcr-arrays). The results were analyzed using Qiagen’s online RT^2^ Profiler PCR Data Analysis software.

#### FACs-based ELISA.

Protein was isolated from snap-frozen kidneys using Tper Buffer (ThermoFisher) according to the manufacturer’s instructions. Protein concentration was determined using Coomassie (ThermoFisher). The isolated protein was analyzed using Biolegend’s Legendplex Mouse Inflammation Panel kit (Biolegend), according to the manufacturer’s instructions. Protein concentrations for the cytokines were then adjusted for total protein in each sample, as previously described (68).

### Statistics

GraphPad Prism 7 (GraphPad) was used to perform all statistical analyses. For analysis of urine biomarker data, comparisons between groups were performed using the Kruskal-Wallis test, and the correlation analysis was performed using Spearman correlation. Receiver operating characteristic (ROC) curve was used to evaluate the performance of ALCAM in discriminating between groups. *P* values less than 0.05 were considered statistically significant. For the animal studies, differences in treatment groups were evaluated using 1-way ANOVA with multiple-comparisons test where *P* < 0.05 was considered statistically significant. Each experiment shown is representative of at least 2 independent experiments.

### Study approvals

#### Human studies.

Informed consent was obtained for each patient for the initial urine collections, and this study was approved by the institutional review boards of all hospitals involved in the study: Department of Gastroenterology, Dermatology and Rheumatology, Johns Hopkins University School of Medicine, Baltimore, Maryland, USA (JHMU cohort); University Hospital Kidney & Liver Clinic, University of Texas Southwestern Medical Center, Dallas, Texas, USA (UTSW cohort); the Division of Rheumatology, Karolinska University Hospital, Stockholm, Sweden (Swedish cohort); the Department of Medicine, Tuen Mun Hospital, New Territories, Hong Kong, China (Hong Kong cohort); the Department of Rheumatology, Renji Hospital, Shanghai, China (Shanghai cohort); and the University of Houston, Houston, Texas, USA.

#### Animal studies.

All animal handling and procedures were approved by the IACUC either at Albert Einstein College of Medicine, New York or University of Houston, Texas.

## Author contributions

SAC and RAR contributed to data collection, data analysis, and study design. SJG, ED, LH, and DC contributed to data analysis. JA contributed to data analysis and study design. TZ, IP, HD, NS, MP, CCM, NJ, IG, and RS contributed data. KRP contributed to study design. SC contributed to study design and analysis. CTN contributed to study design and analysis and wrote the paper. CM and CP contributed data, contributed to study design and analysis, and wrote the paper. The order of the co–first authors was determined based on the extent and duration of the work each contributed to the project.

## Supplementary Material

Supplemental data

## Figures and Tables

**Table 1:**
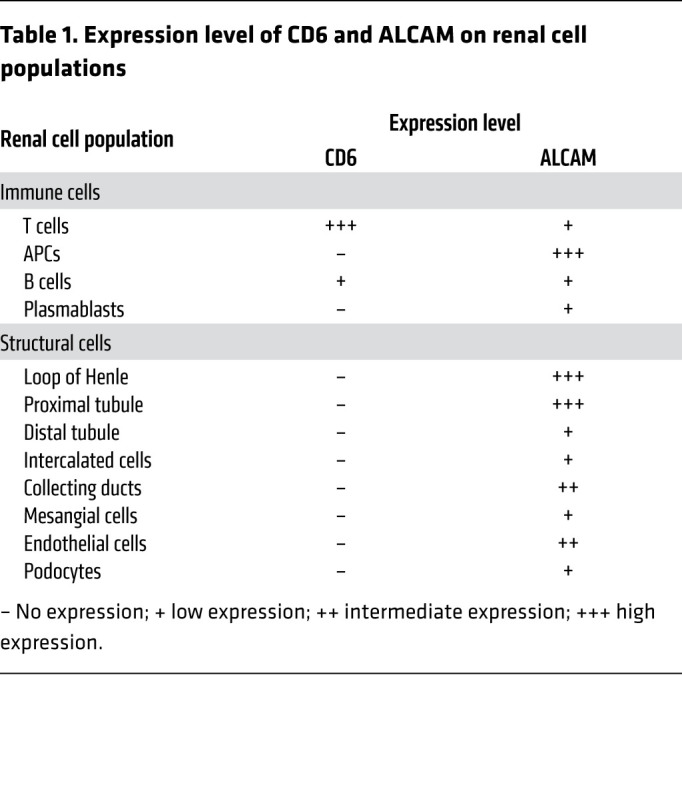
Expression level of CD6 and ALCAM on renal cell populations

## Version 1 01/04/2022

Electronic publication
