# Supplementary material for: The CD6/ALCAM pathway promotes lupus nephritis via T cell–mediated responses
Source: J Clin Invest. 2022 Jan 4;132(1):e147334. doi: 10.1172/JCI147334 (PMC8718154; doi:10.1172/JCI147334)
Supplement: Supplemental data [file jci-132-147334-s043.pdf]

SUPPLEMENTARY INFORMATION

Supplementary Methods

Human Cohorts and ALCAM Quantitation

The human urine biomarker data shown was derived from archived urine samples obtained from five different cohorts, including the Department of Gastroenterology, Dermatology and Rheumatology, Johns Hopkins University School of Medicine, Baltimore, MD, USA (JHMU cohort), University Hospital Kidney & Liver Clinic, University of Texas Southwestern Medical Center, Dallas, TX, USA (UTSW cohort), the Division of Rheumatology, Karolinska University Hospital, Stockholm, Sweden (Swedish cohort), the Department of Medicine, Tuen Mun Hospital, New Territories, Hong Kong, China (Hong Kong cohort), and the Department of Rheumatology, Renji Hospital, Shanghai, China (Shanghai cohort), and included both published datasets (31-33), as well as newly examined patient cohorts. A subset of the patients from the JHMU cohort, and all patients from the Hong Kong cohort, the Swedish cohort, and the Shanghai Cohort (and their urine ALCAM levels), and their detailed demographics have been recently described elsewhere (32-34). When combining previously published datasets, each ALCAM value was normalized by the average ALCAM of healthy controls in each cohort before further analysis in order to minimize any batch effects. In addition to the urine from the recently published studies (32-34), additional urine samples were assayed for the current report, drawing from the UTSW cohort and a second JHMU cohort. Because of the addition of new urine samples in this study, the data shown for each ethnic group includes a larger number of patient samples, relative to the numbers of samples used

in the originally reported studies (32-34; Supplementary Table S1). The demographic information pertaining to the newly added subjects is summarized in Supplementary Table S2.

All patients fulfilled the 2012 Systemic Lupus International Collaborating Clinics (SLICC) criteria for SLE (60). Clinical data were obtained at each sample collection visit, including systemic lupus erythematosus disease activity index (SLEDAI), as described (32, 61). Also documented was the "rSLEDAI or "renal-SLEDAI", which refers to the sum of the scores for the 4 renal criteria in SLEDAI. The active renal group included urine from patients with rSLEDAI≥4. The active non-renal group included urine from patients with SLEDAI≥6 and rSLEDAI=0, and the inactive SLE groups included urine from those with SLEDAI≤4 and rSLEDAI=0. The composition of patients from the different cohorts included in this study is displayed in Supplementary Table S1. Age and ethnicity matched healthy volunteers were also included as controls. For all studies, clean-catch midstream urine samples were collected and stored at -80 °C until the assay. Patient race/ethnicity was ascertained by the investigator (at the Division of Rheumatology, Department of Medicine Solna, Karolinska Institute and Department of Gastroenterology, Dermatology and Rheumatology, Karolinska University Hospital, Stockholm, Sweden, at the Division of Rheumatology and Department of Microbiology & Immunology, Albert Einstein College of Medicine, Bronx, NY, USA, and at Shanghai Jiao Tong University School of Medicine, Shanghai, China) or volunteered by the subject and verified by the investigator (at the Division of Rheumatology, Johns Hopkins University School of Medicine, Baltimore, Maryland, USA, at the Tuen Men Hospital, Hong Kong, PRC, and at the Division of Nephrology, University of Texas Southwestern Medical Center at Dallas, Dallas, TX, USA).

uALCAM levels in all samples and studies (31-33) were measured and normalized using the same method. Specifically, urinary ALCAM levels were measured in urine samples by ELISA using human ALCAM ELISA kit (DY656) from R&D Systems (Minneapolis, MN, USA) according to the manufacturer's instructions. All urine samples were diluted 1:50. Urinary

ALCAM levels were normalized by urine creatinine levels. Urine creatinine levels were measured by the Creatinine Parameter Assay Kit (KGE005, R&D Systems, Minneapolis, MN).

Quantitation of TNF-α and IFN−γ in urine

Urine samples from the cohort derived at UT Southwestern Medical Center described previously ((32), Supplementary Table S1). 23 human subjects (7 active LN, 8 inactive SLE, 8 healthy controls, all female, age range 23–42 years) were interrogated for the levels of 1129 distinct human proteins using a pre-fabricated aptamer-based-targeted proteomic assay (32). The interrogated proteins included ALCAM, IFN-γ, and TNF-α. Obtained values were normalized to urine creatinine.

Quantitation of CD318 in serum and urine

Matched urine and serum samples from LN, inactive SLE, and active non-renal patients were obtained from the Albert Einstein College of Medicine. Informed consent was obtained for each patient for the urine and serum collections, and the study was approved by the institutional review board at Albert Einstein College of Medicine. Clinical data were obtained with each sample collection. For the LN group, samples were obtained within 2 weeks of a renal biopsy sample showing active LN. Inactive disease was defined as SLEDAI <=2, while active non-renal disease was defined as a SLEDAI >=6 and a rSLEDAI=0. Patient characteristics are summarized in Supplementary Table S3.

Urine and serum CD318 levels were measured using the Human CDCP1 DuoSet ELISA (R&D Systems, Minneapolis, MN). Assay was performed according to the manufacturer's

instructions. Serum was diluted 1:100 and urine was diluted 1:20 in assay buffer. These dilutions were determined from pilot experiments examining the optimal dilution to prevent inhibition of detection due to the matrix (i.e. serum, urine). Concentration of soluble CD318 in serum and urine samples were interpolated from a standard curve using a four-parameter logistic (4PL) curve fit.

CD318 co-stimulation of PBMC

48 well plates were coated with one of three stimulation conditions: (1) anti-CD3 monoclonal antibody (mAb) alone, (2) anti-CD3 mAb and ALCAM-Fc, (3) anti-CD3 mAb and CD318-Fc; uncoated wells were used for unstimulated controls. The coating condition for each stimulation condition was as follows: each well was incubated with 0.125µg/ml anti-CD3 (clone UCHT1, BioLegend) and 102.04 uM of either ALCAM-Fc (7187-AL-100, R&D Systems) or CD318-Fc (10402-CU-050, R&D Systems) in 200µl PBS on 48-well plates for 2 hrs at 37°C. Molar concentration for ALCAM-Fc and CD318-Fc was used to ensure that plates were coated with the equivalent number of molecules. After incubation, the plates were washed once with PBS, and then blocked for 30 min with 1% BSA at 37°C followed by two additional washes with PBS.

PBMCs were labeled with Cell Trace Violet (Thermo Fisher) according to manufacturer's instructions, seeded at $2x10^{5}$ cells in a total of 500uls into the coated 48-well plate, and then incubated at 37°C for 72hrs. After 72 hours, supernatant was collected for cytokine detection. Cells were collected and stained with Zombie NIR Fixable Viability Dye (Biolegend, San Diego, CA) according to manufacturer's instructions, and then blocked with human FcR Blocking Reagent (Miltenyi Biotec) for 10' on ice. Cells were stained with CD4-APC (RPA-T4), CD8-BV570 (RPA-T8), CD45RO-AF700 (UCHL1) and CD45RA-BV510 (HI100) to assess naïve and memory

subsets, and CD25-PECy7 (BC96), CD69-BV785 (FN50), CD71-FITC (CY1G4), PD1-BV711 (EH12.2H7), CD40L-BV650 (24-31) and Tim3-BV605 (F38-2E2) to assess activation and proliferation. All fluorescently-conjugated antibodies were purchased from BioLegend (San Diego, CA). Cells were stained for 30’ on ice, before washing and data acquisition on a Cytek Northern Lights instrument. Resulting data was analyzed with FlowJo^{TM} data software.

RNASeq data analysis

Data from single-cell RNA sequencing (scRNA-seq) profiles of clinically indicated renal biopsies obtained from patients with active LN were acquired through the Accelerated Medicines Partnership (AMP) (35). Patient characteristics are contained in Supplementary Table S4. All the scRNA-seq data were analyzed collectively. Cell type identification and clustering was performed with principal component analysis and t-Distributed Stochastic Neighbor Embedding (tSNE) using the Seurat package (version 2.2.1) for R. Briefly, the count matrices were depth-normalized to 10,000 reads and used to identify the set of genes that was most variable across datasets. Ubiquitously expressed genes, such as mitochondrially encoded proteins, were excluded for clustering and removed after variable genes were identified. After clusters were established with tSNE, cell types were identified by examination of expression profiles within each cluster (62).

Supplementary Tables

Supplementary Table S1. A summary of the human urine samples used for uALCAM measurement, parsed by patient cohort, ethnicity and clinical disease status.

	Whites				Asian				African Americans				Hispanic				TOTAL
	**HC**	**Inactive**	**ANR**	**AR**	**HC**	**Inactive**	**ANR**	**AR**	**HC**	**Inactive**	**ANR**	**AR**	**HC**	**Inactive**	**ANR**	**AR**	
JHMU cohort (32)	7	28		13					13	19		14					94
Hongkong cohort (32)					53	80	67	80									280
Swedish cohort (33)	99	45	35	31													210
Shanghai cohort (34)					28	63	59	96									246
UTSW cohort	11	6		12					10	6		35	14	10		37	141
2nd JHMU cohort									15	15	9	28					67
**TOTAL number of patients**	**117**	**79**	**35**	**56**	**81**	**143**	**126**	**176**	**38**	**40**	**9**	**77**	**14**	**10**		**37**	1038

Number in parenthesis indicates references; other numbers in the table indicate the numbers of patients in each group. Blank cells indicate there were no patients in those corresponding groups. HC, healthy control; ANR, active non-renal; AR, active renal.

Supplementary Table S2. **Characteristics of newly included subjects not previously reported**

	HC<br>n=50	Inactive SLE<br>n=37	ANR<br>n=9	AR<br>n=112
Age, yrs	31.94 ± 6.99	35.14 ± 10.83	43.56 ± 8.34	32.02 ± 10.69
Female	33 (66.0%)	33 (89.2%)	9 (100.0%)	99 (88.4%)
Race/Ethnicity				
White	11 (22.0%)	6 (16.2%)	0 (0.00%)	12 (10.7%)
Black	25 (50.0%)	21 (56.8%)	9 (100.00%)	63 (56.3%)
Hispanic	14 (28.0%)	10 (27.0%)	0 (0.00%)	37 (33.0%)
SLEDAI	/	1.54 ± 1.64	6.89 ± 1.45	10.84 ± 5.94
rSLEDAI	/	0 ± 0	0 ± 0	6.96 ± 3.38
UPCR, mg/mg	/	0.16 ± 0.12	0.07 ± 0.06	2.87 ± 3.81
Prednisone	/	20 (54.1%)	3 (33.3%)	82 (73.2%)
Hydroxychloroquine	/	27 (72.9%)	8 (88.9%)	63 (56.3%)
Immunosuppressants	/	22 (59.5%)	6 (66.7%)	66 (58.9%)

Data is presented as average ± standard deviation or number (percentage).
HC, healthy control; ANR, active non-renal; AR, active renal

Supplementary Table S3. Characteristics of subjects tested for soluble CD318.

	**LN (n =40)**	**Non-LN (n =17)**	**Inactive SLE (n=20)**
Age, years	34 ± 10	38 ± 13	43.6 ± 13
Female	36 (90.0%)	16 (94.1%)	15 (75.0%)
Race/Ethnicity			
Black	13 (32.5%)	5 (29.4%)	6 (30.0%)
White	2 (5.0%)	0	0
Asian	0	0	1 (5.0%)
Not reported	9 (22.5%)	4 (23.5%)	8 (40.0%)
Hispanic ethnicity	23 (57.5%)	12 (70.5%)	13 (65.0%)
SLEDAI	8.2 ± 4	6.5 ± 2	1.5 ± 1.1
dsDNA IU/mL	112.6 ± 113.9	97.8 ± 78.3	54.2 ± 70.69
C3 mg/dL	88.6 ± 32	100.6 ± 34.9	123.4 ± 22.2
C4 mg/dL	17.76 ± 8.0	18.08 ± 8.4	22.7 ± 6.4
Hydroxychloroquine	28 (70.0%)	11(64.7%)	18 (90.0%)
Mycophenolate mofetil	20 (50.0%)	2 (11.8%)	4 (20.0%)
Azathioprine	4 (10.0%)	6 (35.3%)	3 (15.0%)
Cyclophosphamide	3 (7.5%)	0	0
Tacrolimus	2 (5.0%)	0	0

Data is presented as average ± standard deviation or number (percentage).

Supplementary Table S4. Characteristics of subjects used for the single cell RNAseq analysis of renal tissue.

	LN Cases (N=24)	Controls (N=9)
Age (mean)	34 ± 11	35 ± 7
Female	21 (87.5%)	6 (66.6%)
Race / Ethnicity		
White	6 (25.0%)	3 (33.3%)
Black	14 (58.3%)	3 (33.3%)
Asian	3 (12.5%)	0 (0%)
Hispanic	5 (20.8%)	1 (11.1%)
Not reported	1 (4.2%)	4 (44.4%)
SLEDAI	13.5 ± 7	N/A
dsDNA, IU/mL	298 ± 407	ND
C3, mg/dL	70 ± 25	ND
C4, mg/dL	11 ± 7.5	ND
Hydroxychloroquine	18 (75.0%)	0
Mycophenolate mofetil	16 (66.7%)	0
Azathioprine	3 (12.5%)	0
Cyclophosphamide	1 (4.2%)	0
Tacrolimus	1 (4.2%)	0
ISN Class		
II	1	N/A
III	5	N/A
IV	9	N/A
V	9	N/A

Supplementary Figures

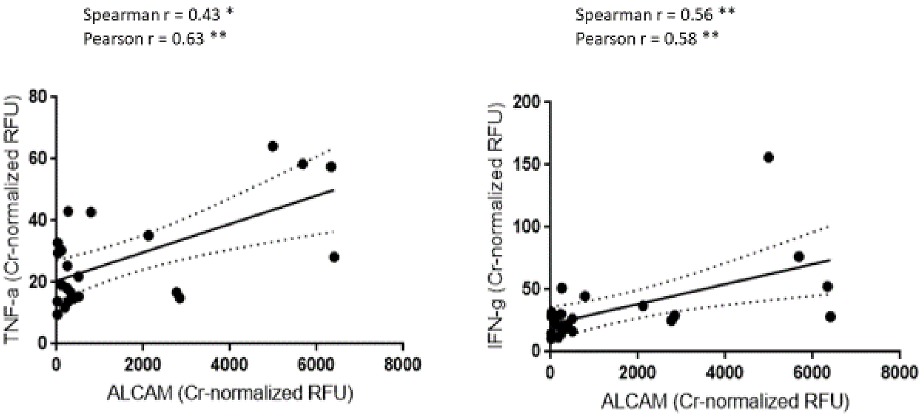

Supplementary Figure 1. Soluble ALCAM in urine (uALCAM) is positively correlated with urine IFN-γ and TNF-α. Urine samples from 23 human subjects (7 active LN, 8 inactive SLE, 8 healthy controls, all female, age range 23–42 years) were interrogated for the levels of 1129 distinct human proteins using an aptamer-based-targeted proteomic assay (32) which included ALCAM, IFN-γ, and TNF-α. Plotted values are urine biomarker levels normalized to urine creatinine. *** p<0.01; * p<0.05.*

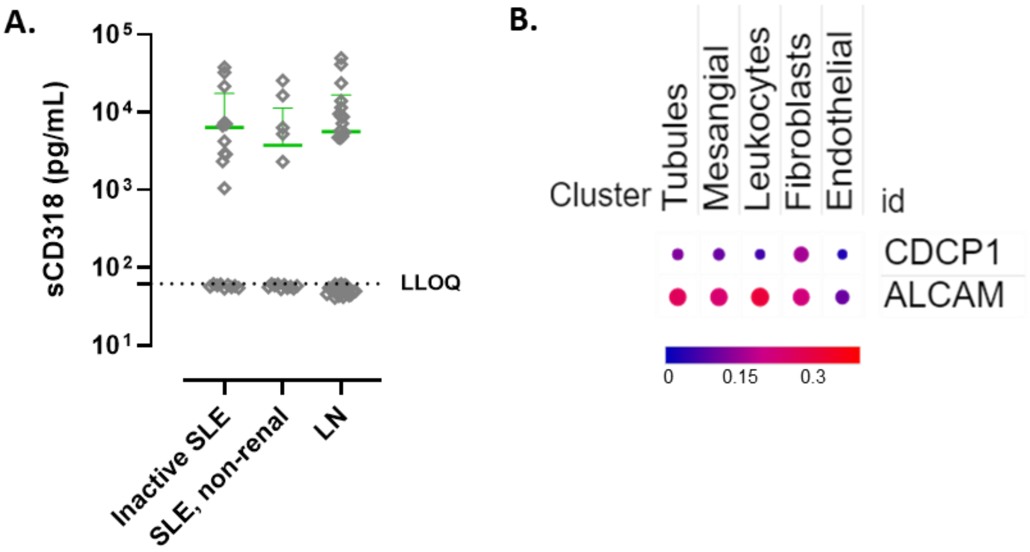

Supplementary Figure 2. CD318 expression in SLE and LN patients. (A) Soluble CD318/CDCP1 levels in the serum of control (n=17), inactive SLE (n=20) and LN (n=40) patients. Data depicted as mean ± 95% CI. (B) CD318/CDCP1 and ALCAM expression on renal cells as assessed using data from single-cell RNA sequencing (scRNA-seq) profiles of clinically indicated renal biopsies obtained from patients with active LN were acquired through the Accelerated Medicines Partnership (AMP). Visualized using the Single Cell Portal of The Broad Institute (https://singlecell.broadinstitute.org/single_cell/study/SCP279/amp-phase-1).

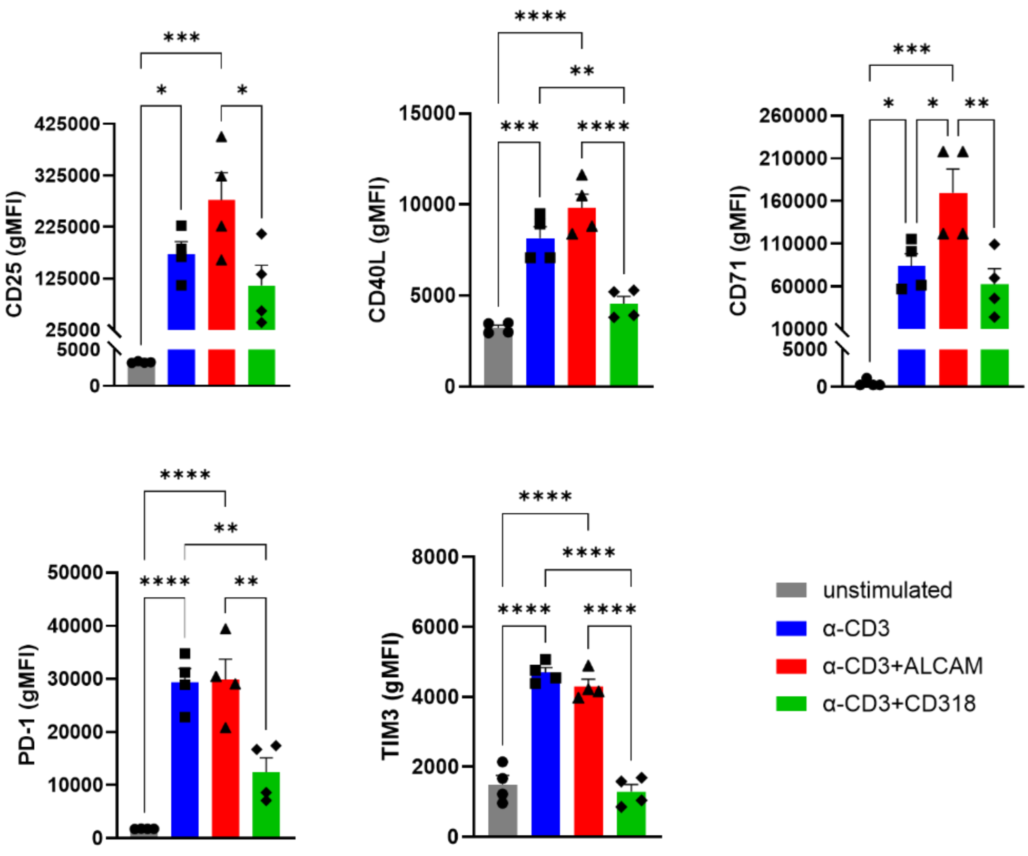

Supplementary Figure 3. Comparison of T cell co-stimulation by ALCAM and CD318. PBMCs were left unstimulated or stimulated using plate-bound anti-CD3 alone, anti-CD3 + recombinant ALCAM-Fc, or anti-CD3+ recombinant CD318-Fc. After 24 hours, cells were collected and analyzed by flow cytometry. T cells were identified using CD3, CD4, CD8, CD45RA, and CCR7. Graphs depict cell surface expression, represented as geometric mean fluorescent intensity (gMFI), of activation markers CD25, CD40L, CD71, PD-1, and TIM3 on CD4 T cells. Data represent mean ± SE. Comparisons between groups were evaluated using one-way ANOVA with multiple-comparisons test. ****p<0.0001; ** p<0.001; ** p<0.01; * p<0.05.*

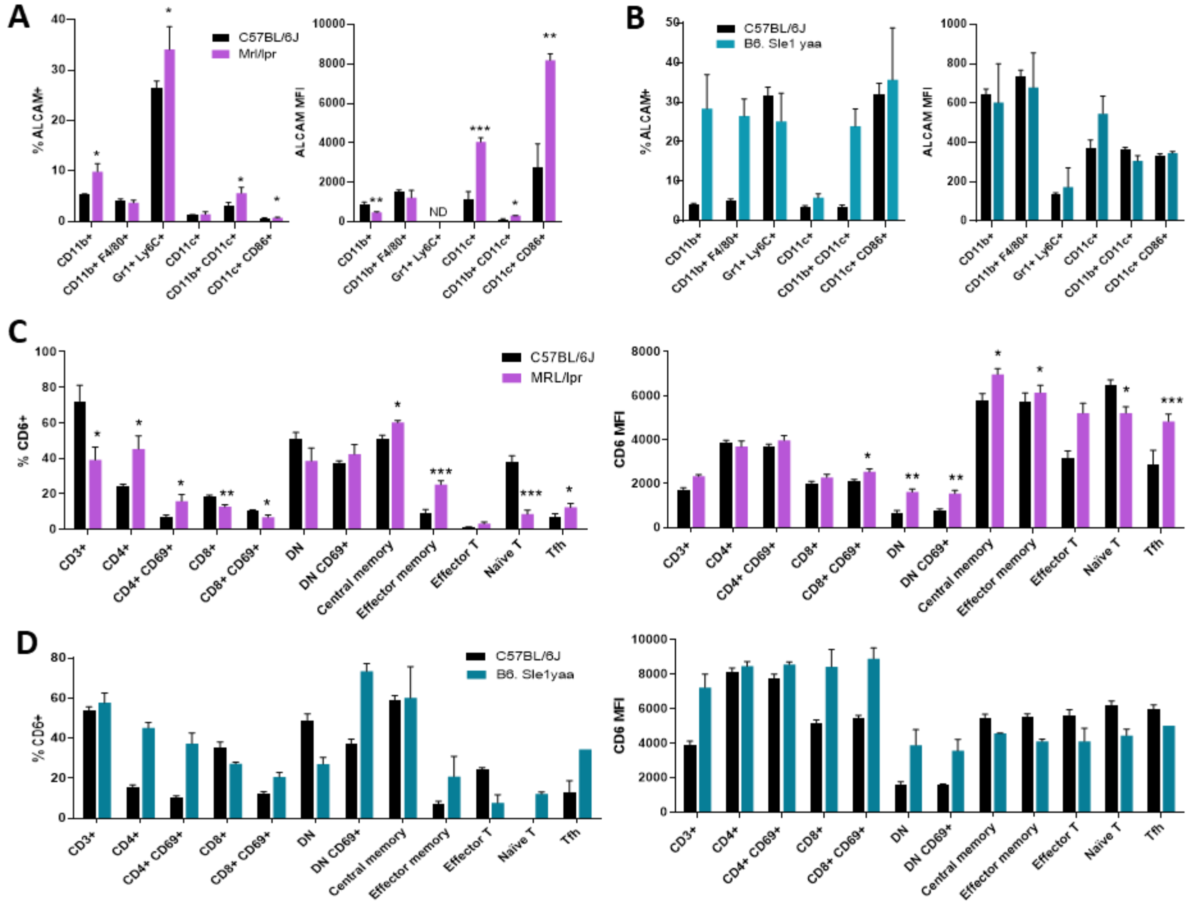

Supplementary Figure 4. Characterization of expression of ALCAM and CD6 on immune cells in the spleen of MRL/lpr and B6.Sle1yaa mice. Splenocytes from C57BL/6J (females, N=8-14, 8-10month old), MRL/lpr (females, N=5-8, 8-month-old) and B6.Sle1yaa (females, N=7, 9-12-month-old) were stained for CD6, ALCAM, and immune cell markers and then analyzed by flow cytometry to determine the CD166 and CD6 expression levels in various immune cell types. (A-B) Comparison of frequency and fluorescence intensity of ALCAM on macrophages, dendritic cells and granulocytes derived from spleens of (A) MRL/lpr vs. C57BL/6J mice or (B) B6.Sle1yaa vs. C57BL/6J mice. (C-D) Comparison of frequency (left) and fluorescence intensity (right) of CD6 on total and activated (CD69+) CD4+, CD8+, and CD4-CD8- T cells, CD4+ central memory (CD62L+CD44+), effector memory, effector (CD62L-CD44+) and naïve T cells (CD62L+CD44-), and Tfh cells (CD45+CD4+CXCR5+PD1+) from (C) MRL/lpr vs. C57BL/6J mice or (D) B6.Sle1yaa vs. C57BL/6J mice. Bar graphs show mean ± SE. Comparisons between groups were done by Mann–Whitney U-test. **** $p<0.001$; ** $p<0.01$; * $p<0.05$*

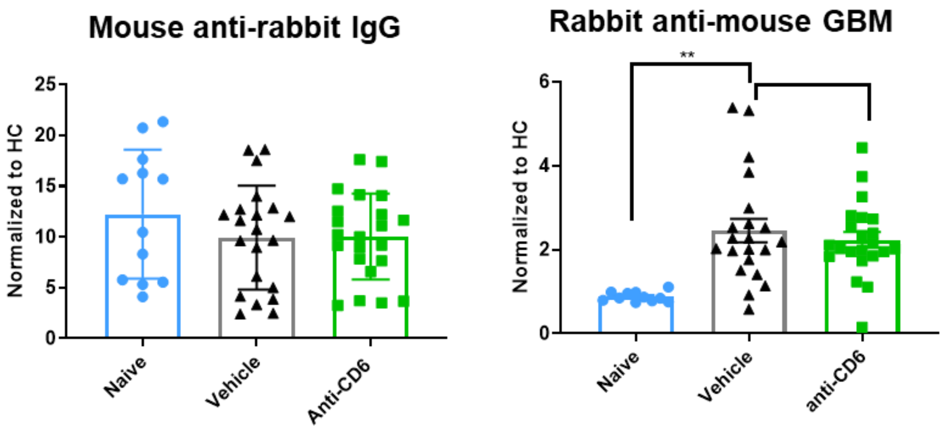

Supplementary Figure 5. Anti-CD6 treatment does not interfere with disease induction in NTN mice. Nephrotoxic serum nephritis (NTN) was induced in female 129/SvJ mice at 10 weeks of age. Mice were immunized with rabbit IgG and CFA on Day 0 to generate mouse anti-rabbit antibodies. At Day 5, mice received nephrotoxic rabbit serum, which then cross-reacted with the mouse anti-rabbit antibodies, causing an antibody-mediated nephritis. Beginning Day 4, mice were treated 3x per week with anti-CD6 (60ug/dose), vehicle control or isotype control. Healthy mice (immunized with rabbit IgG, but not given nephrotoxic serum) were also included as a non-disease control. To ensure that treatment with anti-CD6 antibodies did not interfere with induction of the NTN model, we measured the disease mediating antibodies. (A) Level of mouse anti-rabbit antibodies generated from the day 0 immunization in terminal serum. All groups, including naïve, are immunized against rabbit IgG which resulted in similar levels in each treatment group. (B) Nephrotoxic serum, containing the nephrotoxic rabbit anti-GBM antibodies, was given to both the anti-CD6 treated and vehicle control groups on day 5, but not the disease naïve control mice. Vehicle control and anti-CD6 treated mice had significantly higher levels over the disease naïve control group, confirming proper induction of NTN. ** $p<0.01$
